# Correction: Climbing since the early Miocene: The fossil record of Paullinieae (Sapindaceae)

**DOI:** 10.1371/journal.pone.0251127

**Published:** 2021-04-29

**Authors:** The PLOS ONE Staff

## Notice of Republication

This article was republished on April 17, 2021, to remove identifying information that was incorrectly included in the "Author Response" attachment in the "Peer Review tab" on their published article. The publisher apologizes for the errors. An incorrect version of Fig 2 was also published in error; the authors have provided a corrected version in the republished article. Please download this article again to view the correct version. The originally published, uncorrected article and the republished, corrected articles are provided here for reference.

## Supporting information

S1 File Originally published, uncorrected article. (PDF)

S2 File Republished, corrected article. (PDF)

## References

[1] Jud NA, Allen SE, Nelson CW, Bastos CL, Chery JG (2021) Climbing since the early Miocene: The fossil record of Paullinieae (Sapindaceae). PLoS ONE 16(4): e0248369. 10.1371/journal.pone.0248369 33826635 PMC8026063

