# Correction: Climbing since the early Miocene: The fossil record of Paullinieae (Sapindaceae)

**DOI:** 10.1371/journal.pone.0270540

**Published:** 2022-06-22

**Authors:** The PLOS ONE Staff

## Notice of republication

This article was republished on April 17, 2021, to remove identifying information that was incorrectly included in the "Author Response" attachment in the "Peer Review tab" on their published article. The publisher apologizes for the errors. An incorrect version of Fig 2 was also published in error; the authors have provided a corrected version in the republished article. Please download this article again to view the correct version.
