# Supplementary material for: Climbing since the early Miocene: The fossil record of Paullinieae (Sapindaceae)
Source: PLoS One. 2021 Apr 7;16(4):e0248369. doi: 10.1371/journal.pone.0248369 (PMC8026063; doi:10.1371/journal.pone.0248369)
Supplement: S2 Appendix — Descriptions of Bohlenia americana, Bohlenia insignis, “Cardiospermum” coloradensis, “Cardiospermum” terminale, and “Serjania” rara. (PDF) [file pone.0248369.s002.pdf]

Supplemental Materials for Jud et al.

New World leaf fossils previously (or tentatively) assigned to Paullinieae (Sapindaceae)

UPDATED DESCRIPTIONS

***Bohlenia americana* (Brown) Wolfe and Wehr**

Locality: Republic site in the Klondike Mountain Formation, northeastern Washington, U.S.A.

Original reference: Wolfe and Wehr (1987)

Description: Petiole insertion is marginal. Lamina is elliptic with medial symmetry and strong basal asymmetry in width. Lamina is lobed and toothed (serrate). The apex is acute and straight. The base is obtuse and concave on one side and rounded on the other side. The primary vein framework is pinnate. There is one basal vein and major secondaries are craspedodromous. Other secondaries are aligned with the sinuses and bifurcate around them. Bifurcating secondary veins become accessory veins in adjacent teeth/lobes or loop into adjacent secondaries. A marginal secondary is present. Secondary veins are irregularly spaced with an inconsistent angle to the midvein. Most secondaries diverge from the midvein at 45-60° with rare veins diverging at approximately 75° from the midvein. Major secondaries are excurrent to less commonly decurrent in attachment. Occasional intersecondaries are preserved, less than one per intercostal area. Higher order venation is not preserved. Teeth are irregularly spaced—absent in the basal portion of the lamina. The number of orders of teeth is unclear as the apical portion of the lamina is damaged. Teeth are concave/concave to straight/straight in shape with pointed apices. Principal vein is present in teeth.

Note: The previous description is based on a single specimen (UCMP 9298). The reader is referred to Wolfe and Wehr (1987) for additional description and discussion. Wolfe and Wehr (1987) also describe and figure fruits in their treatment. The fossils, predominately the fruits, are also discussed in: Meyer and Manchester (1997), Manchester (2001), and McClain and Manchester (2001).

***Bohlenia insignis* (Lesquereux) Wolfe and Wehr**

Locality: Joseph Creek, British Columbia, Canada

Original reference: Wolfe and Wehr (1987)

Note: See discussion in Wolfe and Wehr (1987)

***"Cardiospermum" coloradensis* (Knowlton) MacGinitie**

Localities: Green River Formation, Colorado, Wyoming, Utah, U.S.A.

Original references: Knowlton [synonomy in question] (1923), Brown (1929, 1934), MacGinitie (1969), Johnson and Plumb (1995)

Description: Leaves are compound with marginally attached leaflets. Overall leaflet shape is elliptic to ovate and leaflets are asymmetric in medial width. Base symmetry in the leaflets varies from symmetric to asymmetric. Leaflets vary from lobed (more frequent) to unlobed (less frequent) and toothed to untoothed. Most specimens have at least a few teeth, but a few lobed specimens lack teeth. The sinuses of many lobed specimens extend nearly to the midvein. Sinuses of lobes may be rounded or angular. Apices are acute and often straight, but less frequently convex. The base angle is acute to rarely obtuse and straight to slightly convex in shape. The primary vein framework of the leaflets is pinnate. Secondaries are craspedodromous when they terminate in a lobe or a tooth, but brochidodromous in areas that lack lobes and teeth. Many specimens preserve a marginal secondary. Secondary veins diverge from the midvein at 35-60° with divergence between 40-50° the most common. Intersecondaries are rarely preserved. Tertiary veins loop before reaching the margin of the leaflets and often intersect the midvein at approximately 90° or slightly less. Higher order venation is not well preserved. Tooth spacing is irregular with one tooth per centimeter when present. Typically, one order of teeth is present, however some specimens preserve two orders. Sinuses are angular in shape and teeth are concave/concave in shape. A principal vein is present in each tooth which terminates at the apex of the tooth or slightly on the distal flank. In some teeth it appears that the primary vein extends beyond the tooth apex. This may be preserved as a little nub past the tooth apex or a darker concentration of tissue.

Specimens examined: Descriptions and figures in the "original references," specimens included in MacGinitie (1969) that are stored in the University of California Museum of Paleontology (UCMP), and other Green River Formation specimens in the UCMP and the Smithsonian Institution (USNH).

***"Cardiospermum" terminale* (Lesquereux) MacGinitie**
[Note: originally published as *C. terminalis*, but nomenclature updated by Becker (1961)]

Localities: Florissant Formation, central Colorado, U.S.A.; Upper Ruby River Basin, southwestern Montana, U.S.A.

Original references: MacGinite (1953), Becker (1961), Manchester (2001)

Description: Leaves once pinnately compound (even); leaflet attachment varies from petiolate to sessile. Leaflets with opposite arrangement and marginal attachment. Leaflet shape is ovate to elliptic with a few specimens obovate if the lobes originate closer to the apex. Leaflets vary from symmetric to asymmetric in both medial and basal width. Leaflets are lobed and vary from toothed to untoothed. In some cases, the sinuses of the lobes extend nearly to or to the midvein. The apex angle is acute; most apices are straight, but some are concave to convex—often concave on one side and convex on the other side. Bases are acute to obtuse and vary from straight to slightly concave to convex. Obvious terminal leaflets have a narrow base with a

straight to concave shape. Rarely, specimens have large lobes originating near the base of the leaflet, resulting in a concavo-convex base shape. The primary vein framework is pinnate and secondaries are craspedodromous. Some secondaries meet the sinus of teeth and/or lobes, whereas others loop along the margin (brochidodromous) where teeth and/or lobes are absent. A marginal secondary is present. Major secondaries are irregularly spaced; secondaries interface with the midvein at angles of 30-55°. The intercostal tertiary vein fabric is reticulate; higher order venation is not preserved. Margin is toothed or untoothed; if toothed there is one order of irregularly spaced teeth with approximately one tooth per centimeter. Sinuses are angular and teeth are concave/concave in shape. A principle vein is present in each tooth.

Specimens examined: Descriptions and figures in the "original references," published and figured specimens available on https://flfo-search.colorado.edu/, and specimens in the collections of Florissant Fossil Beds National Monument.

***"Serjania" rara* MacGinitie**

Localities: Blue Rim site in the Bridger Formation, southwestern Wyoming, U.S.A.; Kisinger Lakes site in the Aycross Formation, northwestern Wyoming, U.S.A.

Original references: Allen (2017), MacGinitie (1974)

Description: Leaves compound with marginally attached petiolate to sessile leaflets. Leaflets are microphyll and are elliptic to ovate in overall shape; occasionally terminal leaflets vary to obovate. Medial symmetry of the leaflets varies from slightly asymmetric to asymmetric—especially in lateral leaflets. The leaflet bases vary from slightly to fully asymmetric in width. Leaflets vary from unlobed to lobed. Margins are toothed, both crenate and serrate. Leaflet apices are acute and straight to convex. Base angle varies from acute to obtuse and the base shape varies from straight to convex to slightly concave. The primary vein framework is pinnate. Secondaries are predominately craspedodromous, but they are sometimes aligned with and bifurcating before reaching a sinus. The bifurcating veins then continue both above and below the sinus. Interior secondaries are absent; marginal secondaries are visible in some, but not all specimens. Secondaries depart midvein at an angle of 40-50°. Intersecondaries are present. The intercostal tertiary vein fabric is likely percurrent; higher order venation is irregular reticulate. Tooth spacing is irregular with one to two orders of teeth (depending on the distinction between what is classified as a lobe vs. a tooth). Where teeth are present, there are 1-2 teeth per centimeter. The sinuses are angular, and the teeth are concave/concave in shape. A principal vein is present in each tooth that terminates at or very close to the apex of the tooth. Many specimens preserve an extension past the apex of the teeth. These may be a continuation of the principal vein or a gland.

Specimens examined: All specimens from the Blue Rim site in the collections of the Florida Museum of Natural History collection (UF) and specimens included in MacGinitie (1974) that are housed in the University of California Museum of Paleontology (UCMP).

REFERENCES

Allen, S. E. 2017. The uppermost Lower Eocene Blue Rim flora from the Bridger Formation of southwestern Wyoming: Floristic composition, paleoclimate, and paleoecology. Ph.D., University of Florida.

Becker, H. F. 1961. Oligocene plants from the Upper Ruby River Basin, southwestern Montana. Geological Society of America.

Brown, R. W. 1929. Additions to the flora of the Green River Formation, 279-299. United States Department of the Interior Professional Paper 154-J, Washington, D.C., USA.

Brown, R. W. 1934. The recognizable species of the Green River flora, 45-68. United States Department of the Interior Professional Paper 185-C, Washington, D.C., USA.

Johnson, K. R., and C. Plumb. 1995. Common plant fossils from the Green River Formation at Douglas Pass, Colorado, and Bonanza, Utah. *In* W. R. Averett [ed.], The Green River Formation in the Piceance Creek and Eastern Uinta Basins, 121-130. Grand Junction Geological Society Grand Junction, CO.

Knowlton, F. H. 1923. Revision of the flora of the Green River Formation, with descriptions of new species. *USGS Professional Paper* 131-F: 133-197.

MacGinitie, H. D. 1953. Fossil Plants of the Florissant Beds, Colorado. Carnegie Institution of Washington Publication 599, Washington, D.C.

MacGinitie, H. D. 1969. The Eocene Green River Flora of Northwestern Colorado and Northeastern Utah. 1-202. University of California Press, Berkeley, California, USA

MacGinitie, H. D. 1974. An Early Middle Eocene flora from the Yellowstone-Absaroka Volcanic Province, northwestern Wind River Basin, Wyoming. 1-103. University of California Publications in Geological Science, Berkeley, California, USA.

Manchester, S. R. 2001. Update on the megafossil flora of Florissant, Colorado. *In* E. Evanoff, K. M. Gregory-Wodzicki, and K. R. Johnson [eds.], Fossil flora and stratigraphy of the Florissant Formation, Colorado, vol. 4, 137-161. Denver Museum of Nature & Science, Denver.

McClain, A. M., and S. R. Manchester. 2001. *Dipteronia* (Sapindaceae) from the Tertiary of North America and implications for the phytogeographic history of the Aceroideae. *American Journal of Botany* 88: 1316-1325.

Meyer, H. W., and S. R. Manchester. 1997. The Oligocene Bridge Creek Flora of the John Day Formation, Oregon. University of California Publicatins in Geological Sciences. University of California Press, Berkeley.

Wolfe, J. A., and W. Wehr. 1987. Middle Eocene Dicotyledonous Plants from Republic, Northeastern Washington. *U.S. Geological Survey Bulletin* 1597: 1-25.
