# Supplementary figures and images for: Climbing since the early Miocene: The fossil record of Paullinieae (Sapindaceae)
Source: PLoS One. 2021 Apr 7;16(4):e0248369. doi: 10.1371/journal.pone.0248369 (PMC8026063; doi:10.1371/journal.pone.0248369)

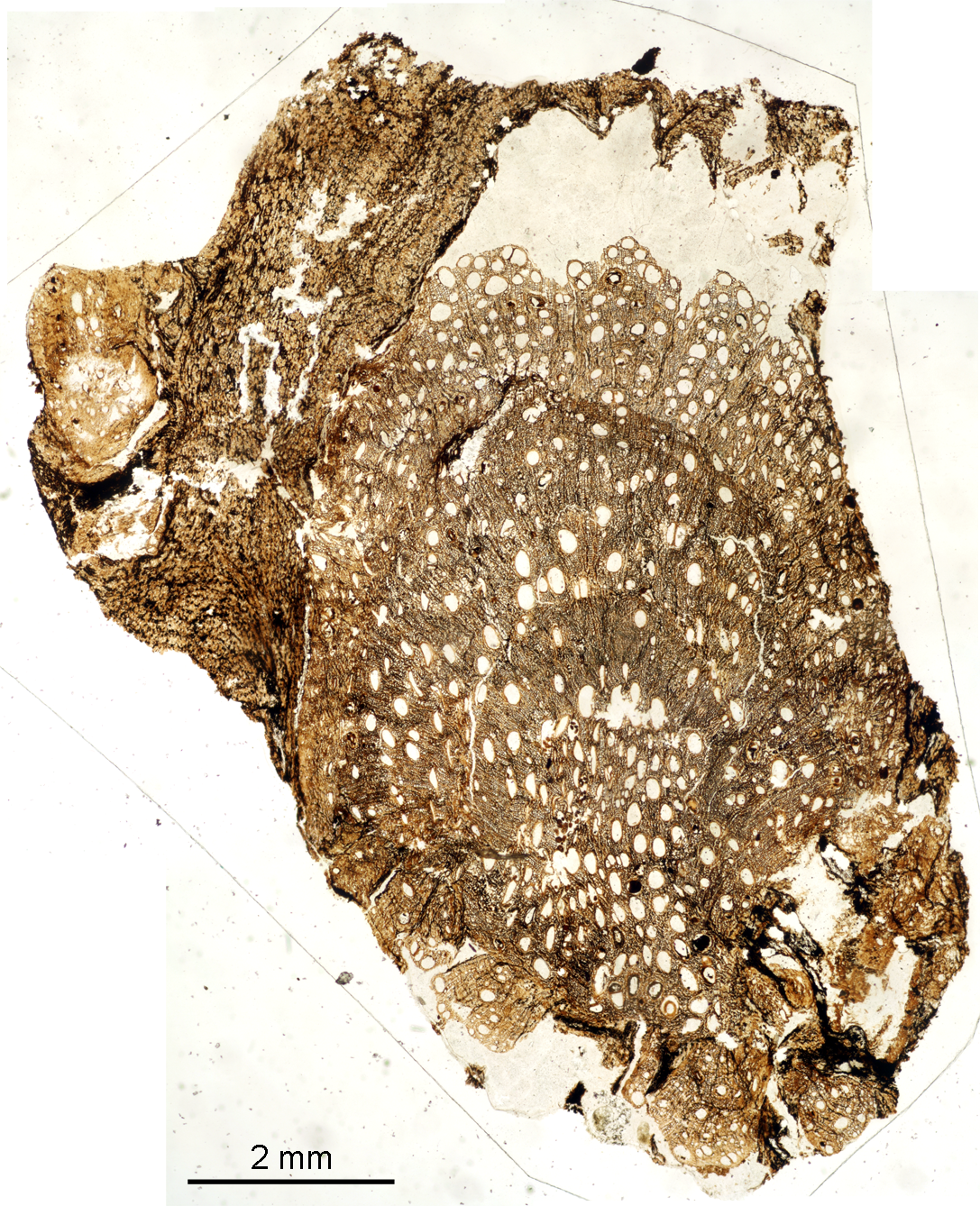

Supplement: S1 Fig — (TIF) [file pone.0248369.s003.tif]

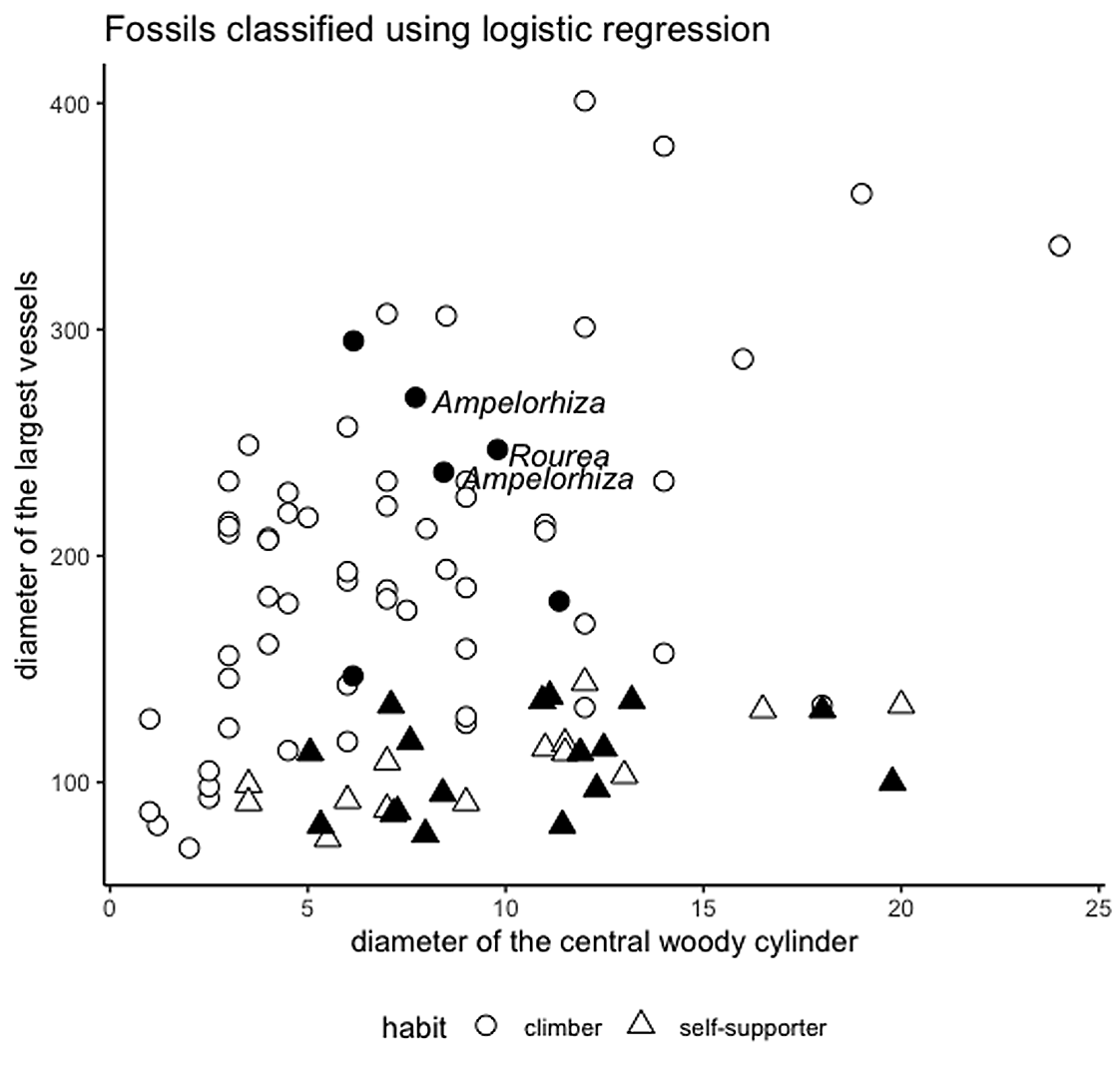

Supplement: S2 Fig — Filled points are fossil axes from the Lirio East site classified as either climbers or self-supporters using logistic regression. We applied a conservative decision threshold of 0.95 for classifying lianas. (TIF) [file pone.0248369.s004.tif]
