# Supplementary material for: Correction: Climbing since the early Miocene: The fossil record of Paullinieae (Sapindaceae)
Source: PLoS One. 2021 Apr 29;16(4):e0251127. doi: 10.1371/journal.pone.0251127 (PMC8084118; doi:10.1371/journal.pone.0251127)
Supplement: S1 File — (PDF) [file pone.0251127.s001.pdf]

PLOS ONE

RESEARCH ARTICLE

Climbing since the early Miocene: The fossil record of Paullinieae (Sapindaceae)

Nathan A. Jud[1,3]*, Sarah E. Allen[2], Chris W. Nelson[3¤], Carolina L. Bastos[4], Joyce G. Chery[5]*

1 Department of Biology, William Jewell College, Liberty, MO, United States of America, **2** Department of Biology, Penn State Altoona, Altoona, PA, United States of America, **3** Florida Museum of Natural History, University of Florida, Gainesville, FL, United States of America, **4** Laboratory of Plant Anatomy, Department of Botany, Instituto de Biociências, Universidade de São Paulo, São Paulo, SP, Brazil, **5** School of Integrative Plant Sciences, Section of Plant Biology and the L.H. Bailey Hortorium, Cornell University, Ithaca, NY, United States of America

¤ Current address: Gainesville, FL, United States of America
* judn@william.jewell.edu (NAJ); jgc235@cornell.edu (JGC)

Check for updates

OPEN ACCESS

Citation: Jud NA, Allen SE, Nelson CW, Bastos CL, Chery JG (2021) Climbing since the early Miocene: The fossil record of Paullinieae (Sapindaceae). PLoS ONE 16(4): e0248369. https://doi.org/10.1371/journal.pone.0248369

Editor: William Oki Wong, Indiana University Bloomington, UNITED STATES

Received: October 16, 2020

Accepted: February 23, 2021

Published: April 7, 2021

Peer Review History: PLOS recognizes the benefits of transparency in the peer review process; therefore, we enable the publication of all of the content of peer review and author responses alongside final, published articles. The editorial history of this article is available here: https://doi.org/10.1371/journal.pone.0248369

Copyright: © 2021 Jud et al. This is an open access article distributed under the terms of the Creative Commons Attribution License, which permits unrestricted use, distribution, and reproduction in any medium, provided the original author and source are credited.

Data Availability Statement: All relevant data are within the paper and its Supporting information files.

Funding: This study received support from the National Science Foundation (NSF) Award Number

Abstract

Paullinieae are a diverse group of tropical and subtropical climbing plants that belong to the soapberry family (Sapindaceae). The six genera in this tribe make up approximately one-quarter of the species in the family, but a sparse fossil record limits our understanding of their diversification. Here, we provide the first description of anatomically preserved fossils of Paullinieae and we re-evaluate other macrofossils that have been attributed to the tribe. We identified permineralized fossil roots in collections from the lower Miocene Cucaracha Formation where it was exposed along the Culebra Cut of the Panama Canal. We prepared the fossils using the cellulose acetate peel technique and compared the anatomy with that of extant Paullinieae. The fossil roots preserve a combination of characters found only in Paullinieae, including peripheral secondary vascular strands, vessel dimorphism, alternate intervessel pitting with coalescent apertures, heterocellular rays, and axial parenchyma strands of 2–4 cells, often with prismatic crystals. We also searched the paleontological literature for other occurrences of the tribe. We re-evaluated leaf fossils from western North America that have been assigned to extant genera in the tribe by comparing their morphology to herbarium specimens and cleared leaves. The fossil leaves that were assigned to *Cardiospermum* and *Serjania* from the Paleogene of western North America are likely Sapindaceae; however, they lack diagnostic characters necessary for inclusion in Paullinieae and should be excluded from those genera. Therefore, the fossils described here as *Ampelorhiza heteroxylon* gen. et sp. nov. are the oldest macrofossil evidence of Paullinieae. They provide direct evidence of the development of a vascular cambial variant associated with the climbing habit in Sapindaceae and provide strong evidence of the diversification of crown-group Paullinieae in the tropics by 18.5–19 million years ago.

0966884. The funders had no role in study design, data collection and analysis, decision to publish, or preparation of the manuscript. No additional external funding received for this study.

Competing interests: The authors have declared that no competing interests exist.

Introduction

Paullinieae (Sapindaceae) are tropical and subtropical woody vines (i.e., lianas), herbaceous climbers (i.e., vines), and seldom shrubs [1]. The six genera of Paullinieae–*Paullinia* L., *Serjania* L., *Cardiospermum* Kunth., *Urvillea* Kunth., *Lophostigma* Radlk., and *Thinouia* Triana & Planch–form a clade [2–4, 21] defined by their tendrilate climbing habit and presence of stipules [21]. With approximately 475 species [21], they comprise nearly one quarter of all species in Sapindaceae. The Paullinieae are one of the four successively nested tribes of the Supertribe Paulliniodae sensu by Acevedo-Rodríguez et al. [21], however the other members–Athyaneae, Bridgesieae, Thouinieae–are all trees and shrubs. Numerous members of Paullinieae undergo developmental re-patterning during the production of secondary xylem (i.e., wood) and secondary phloem (i.e., inner bark), resulting in the formation of "vascular cambial variants," such as continuous or discontinuous successive cambia, neoformations forming peripheral secondary vascular strands (i.e., corded [5]), compound stems, fissured xylem, divided xylem, lobed xylem, and phloem wedges [5–19].

The monophyly of Paullinieae within the subfamily Sapindoideae is supported by morphology [20] and molecular sequence data [2–4, 21, 22]. Molecular phylogenetic analyses have repeatedly yielded a long branch subtending the Paullinieae [2–4], suggesting shifts in nucleotide substitution rates potentially associated with the evolution of the climbing habit. Previous efforts to calibrate the phylogeny of Sapindaceae have yielded Oligocene or Miocene estimates for the age of crown-group Paullinieae [23–25]; however, critical evaluation of the fossil record is necessary to constrain the timing of diversification and the evolution of morphology and anatomy of Paullinieae.

Although the fossil record of Sapindaceae is rich e.g., [1, 26], macrofossils of Paullinieae are rare and at least some previous identifications are unreliable. Here, we describe the first anatomically preserved macrofossils of Paullinieae. The fossils are roots, but nonetheless provide strong evidence of the climbing habit based on wood anatomy associated with climbing in Sapindaceae. Next, we evaluate fossil leaves that have been attributed to the tribe. Then, we summarize the fossil record of the tribe with a focus on macrofossils and identify occurrences best suited for calibrating time-trees [27]. Finally, we discuss the implications of our findings for future studies of the evolution of Paullinieae.

Materials and methods

Geologic setting

Two fossil roots were identified in a collection from the Lirio East site in lower part of the Cucaracha Formation along the Culebra Cut (Gaillard Cut) of the Panama Canal (Fig 1). These collections were made in in 2007 by F. Herrera and S.R. Manchester. The lower Cucaracha Formation consists of deltaic and coastal swamp deposits laid down during the early Miocene when the nearby Pedro-Miguel Volcanic Complex was active [28–31]. At the Lirio East site, fossil fruits as well as woods with bark are preserved as calcareous permineralizations in a poorly sorted, carbonate-cemented sandstone [32].

So far, remains of *Sacoglottis* (Humiriaceae) [33], *Oreomunnia* (Juglandaceae) [34], *Parinari* (Chrysobalanaceae) [35], *Mammea* (Calophyllaceae) [36], *Rourea* (Connaraceae) [37], and *Spondias* (Anacardiaceae) [38], have been described. Plant macrofossils from elsewhere in the Cucaracha Formation include palm stem fragments [39], *Guazuma*-like Malvaceae [40], legume woods [39, 41], and a Malpighialean wood [42]. Fossil pollen from the Cucaracha Formation includes at least 52 pollen types [43]. Together, these records suggest the vegetation was primarily tropical rainforest near the paleoshoreline of central Panama [43].

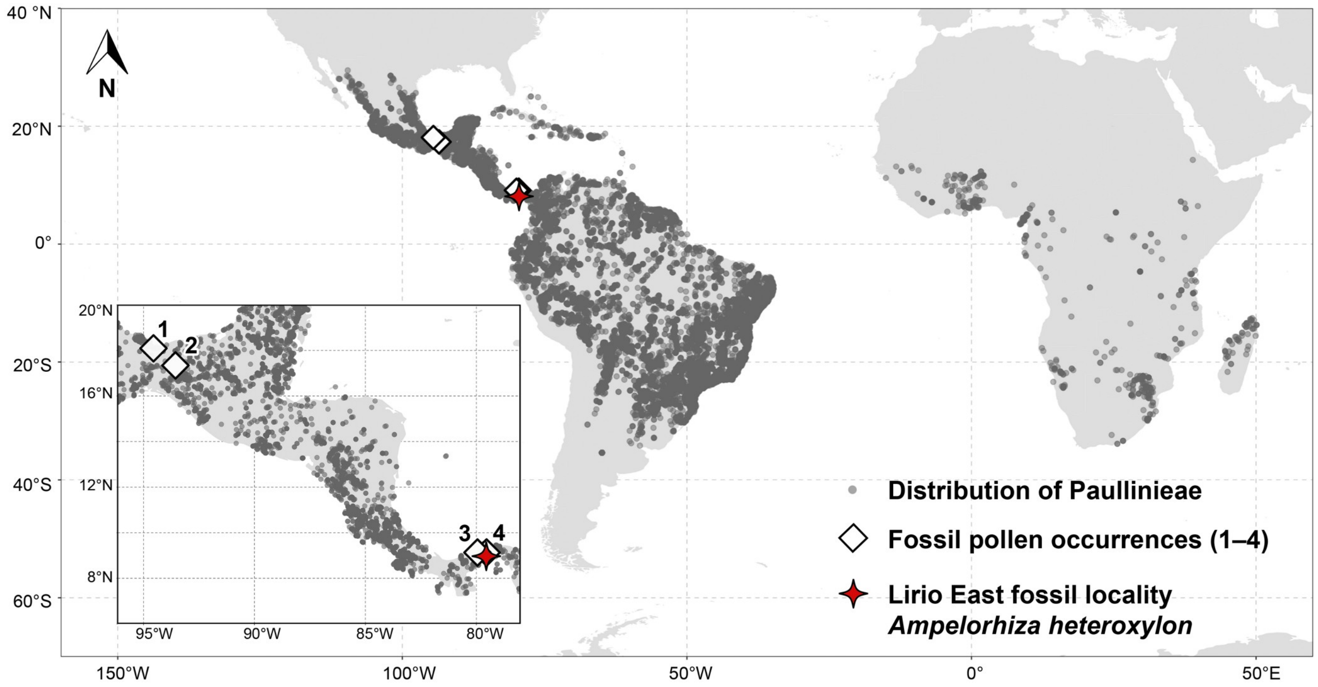

Fig 1. Native distribution of Paullinieae and fossil occurrences. Modern occurrence data from the BIEN database [45, 46]. Red star indicates the location of the Lirio East fossil site where the fossil roots were collected. Fossil pollen occurrence codes: 1 = *Serjania* sp., upper Miocene Paraje Solo Formation [47–49]; 2 = *Serjania* sp. and *Paullinia* sp., lower-middle Miocene Méndez Formation [50]; 3 = *Serjania* sp. and *Paullinia* sp., upper Miocene Gatun Formation [49, 51]; 4 = *Serjania* sp., *Paullinia* sp., and *Cardiospermum* sp., upper Eocene Gatuncillo Formation [48, 52] Occurrence data were extracted from BIEN ver. 4.1 database using the RBIEN package [46], supplemented with *C. pechuelii* data from GBIF [53]. *Cardiospermum* spp. distribution data follows native ranges determined by [54, 55] (excluding controversial range in India).

https://doi.org/10.1371/journal.pone.0248369.g001

Fossil preparation

We cut the fossils in transverse and tangential and radial longitudinal sections using a Microslice 2 annular saw and prepared serial sections using the cellulose acetate peel technique [44]. Peels were mounted on 25 x 75 mm glass slides with Canada Balsam or Eukitt mounting medium and examined using light microscopy. Images of microscopic features were captured with a Canon EOS digital camera mounted on a Nikon compound microscope with transmitted light and processed with Adobe Photoshop (San Jose, California, USA). All specimens, peels, and microscope slides are curated at the Florida Museum of Natural History Paleobotanical Collections, Gainesville, Florida, United States.

Terminology and measurement protocols for the wood anatomy generally follow the IAWA Hardwood List [56] but we adapted our approach for characters particular to Paullinieae [64]. Summary statistics for anatomical characters were calculated from 25 measurements. The fossil exhibits vessel dimorphism; this term has been used for both highly skewed distributions and bimodal distributions [57–59], so we measured all vessels in the central xylem cylinder [14] of a single transverse peel (n = 162) from the holotype (UF 19391-63016) to generate a histogram of the distribution of vessel diameters. Then, we used the densityMclust function in the package mclust [60] in R [61] to identify the modes in the distribution that correspond to the narrow and wide vessel classes. We report "narrow vessel diameter" and "wide vessel diameter" as two separate characters. All measurements were made in ImageJ 1.50a [62].

Table 1. Summary of pre-Quaternary macrofossils that have been assigned to Paullinieae.

Species	Organ	Formation	Age	Country	References	Status
Ampelorhiza heteroxylon	root	Cucaracha	Mi.	Panama	This study	accepted
Bohlenia spp.	leaf	Klondike Mountain	Eo.	USA	[85, 86]	rejected
"Cardiospermum" coloradensis	leaf	Green River	Eo.	USA	[81]	rejected
"Cardiospermum" terminale	leaf	Florissant; Renova	Eo.	USA	[77, 78, 95]	rejected
"Serjania" rara	leaf	Aycross; Bridger	Eo.	USA	[74, 75]	rejected
Serjania mezzalire	leaf	Rio Claro	Ol.	Brazil	[89]	uncertain
Serjania itaquaquecetubensis	leaf	Itaquaquecetuba	Mi.	Brazil	[87]	uncertain
Serjania laceolata	leaf	Itaquaquecetuba	Mi.	Brazil	[87]	uncertain

Each identification is classified as accepted, rejected, or uncertain (material is consistent with Paullinieae, but alternative interpretations have not been ruled out). Mi.: Miocene, Ol.: Oligocene, Eo.: Eocene. See text for further justification of status.

https://doi.org/10.1371/journal.pone.0248369.t001

Fossil leaves

We searched the literature for fossils identified as Paullinieae (Table 1). Of the species we found, we examined specimens and images for those from North America and we re-described their morphology following the format of the Manual of Leaf Architecture [63]. For putative occurrences from South America and Europe, we evaluated images and descriptions from the published literature. We used herbarium collections and online images to survey angiosperm families for leaves with organization, margin type, and venation patterns similar to the fossil leaf taxa re-described here (originally assigned to modern genera within Paullinieae). Then, we compared the morphology of the fossils with leaves from extant genera in Paullinieae and with leaves of selected genera outside Sapindaceae that exhibit similarities in organization, shape, margin, and venation patterns. Cuticle was not preserved on any of the fossil leaves we examined and we did not evaluate cuticle for diagnostic characters. Comparisons are based on dried specimens in the University (UC) and Jepson (JEPS) Herbaria at the University of California —Berkeley, the R. L. McGregor Herbarium (KANU) at the University of Kansas, images available online via JSTOR Global Plants, and cleared and stained leaves in the National Cleared Leaf Collection (NCLC-H; https://collections.peabody.yale.edu/pb/nclc/).

Phylogenetic analysis

We obtained the concatenated multiple sequence alignments from [21] and [22]. From these datasets, we exclusively selected species within the supertribe Paullionieae as described by Acevedo-Rodríguez et al. [21], which includes Athyaneae, Bridgesieae, Thouinieae, and Paullinieae, totalling 100 ITS and 88 *trnL* intron sequences from [21], and 115 ITS sequences from [22]. We then combined the two ITS datasets and realigned them in Geneious Prime 2021.0.3 (https://www.geneious.com) using the MUSCLE v3.8.425 aligner under default settings; the *trnL* intron sequences were realigned under the same settings.

We then obtained wood anatomy data for 11 terminals from [13] and 33 terminals from [64], and one terminal from [20], now available on morphobank (morphobank.org/permalink/?P3910), and scored the fossil for 22 out of the 27 anatomy characters. Finally, we added the character "habit" (0 = self-supporter, 1 = climber) and scored it for all extant species. Although the wood anatomy characters scored for extant species were observed in stems and the fossils are roots, available evidence indicates that differences in wood anatomy between stems and roots within individual plants tend to be quantitative rather than qualitative [16, 65,

66]. The resulting dataset (S1 Appendix) comprises 216 tips and 1517 characters with three partitions: anatomy (1-28), ITS (29-882), and *trnL* intron (883-1517).

We estimated the phylogenetic position of the fossil taxon using a Bayesian analysis with two runs each of four chains (three hot, one cold, temp = 0.02) in MrBayes 3.2.7 [67]. We applied the GTR+G model of nucleotide evolution to the ITS and *trnL* intron partitions. The Mk model with rates drawn from a lognormal distribution was applied to the anatomy partition. The analysis ran for 12 million generations, sampling trees every 1000th generation. The analysis converged with a standard deviation of split frequencies of 0.007428 and the estimated sample size (ESS) of all parameters exceeded 2108. All trees were generated using the post burnin (25% of initial trees discarded) from the combined MrBayes runs. The allcompat consensus tree (50% majority rule consensus with compatible groups added) was generated with the MrBayes command: contype = allcompat and annotated using iToL v4 [68]. The maximum clade credibility (MCC) tree was generated with Tree Annotator v1.10.4 [69], and the maximum a posteriori tree (MAP) was generated with RevBays v1.10 [70]. The MrBayes input nexus file (data matrix), allcompat consensus, MCC, and MAP trees, and full accession list with associated molecular and anatomical data references are provided in (S1 Appendix).

Nomenclature

The electronic version of this article in Portable Document Format (PDF) in a work with an ISSN or ISBN will represent a published work according to the International Code of Nomenclature for algae, fungi, and plants, and hence the new names contained in the electronic publication of a PLOS ONE article are effectively published under that Code from the electronic edition alone, so there is no longer any need to provide printed copies. The online version of this work is archived and available from the following digital repositories: PubMed Central and LOCKSS.

Results

Fossil roots

Family. Sapindaceae Jussieu.

Subfamily. Sapindoideae Burnett.

Tribe. Paullinieae (Kunth) DC.

Genus. *Ampelorhiza* Jud, S.E. Allen, Nelson, Bastos & Chery gen. nov.

Generic diagnosis. Roots woody with neoformations forming peripheral secondary vascular strands; vessels of two distinct size classes, wide vessels solitary and in tangential multiples, narrow vessels in long radial multiples; intervessel pits alternate with slit-like coalescent apertures on the walls of large vessels; heterocellular rays composed of mixed upright, square, and procumbent cells; axial parenchyma strands 2–4 or more cells tall, often chambered with prismatic crystals.

Type species. *Ampelorhiza heteroxylon* Jud, S.E. Allen, Nelson, Bastos & Chery gen. et sp. nov.

Specific diagnosis. As for genus.

Holotype. UF 19391-63016 (Figs 2 and 3).

Paratype. UF 19391-63026 (S1 Fig).

Repository. Florida Museum of Natural History (FLMNH), Gainesville, Florida, U.S.A.

Type locality. Panama; Culebra Cut, northeast side of the Panama Canal (N 9.051375˚, W 79.649027˚, WGS84).

Stratigraphic position and age. Cucaracha Formation; early Miocene, c. 18.5–19 Ma [30, 31].

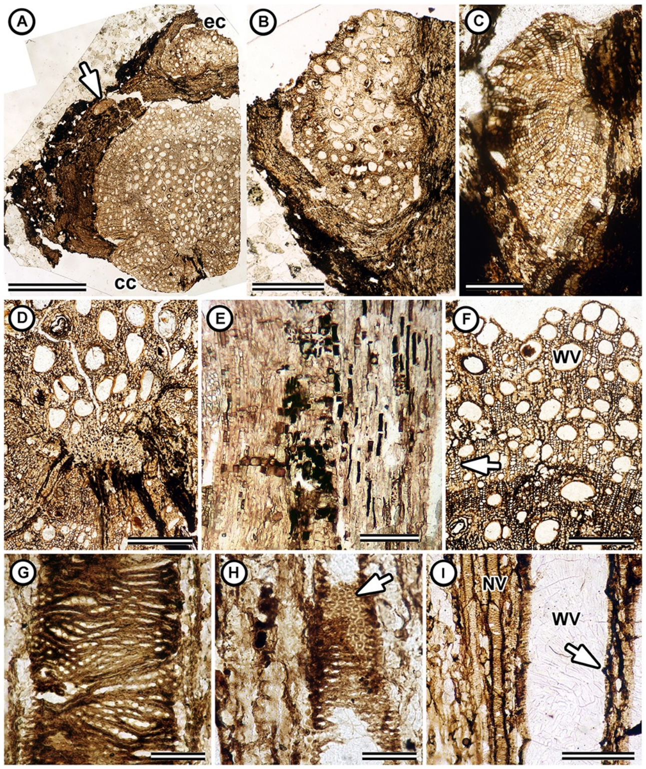

Fig 2. Cambial variant and vessel characters in *Ampelorhiza heteroxylon*. (A) Transverse section of the stem showing diffuse-porous wood of the central cylinder (cc) and peripheral vascular strands (ps) in the external tissues. Arrow indicates the position of the smaller of two peripheral vascular cylinders. Specimen UF 19391-63016 XS peel 10. (B) Close up transverse section of the larger of two peripheral vascular strands. Specimen UF 19391-63016 XS peel 10. (C) Transverse section of the smaller of two peripheral vascular cylinders. There is no pith. Specimen UF 19391-63016 XS peel 10. (D) Close up of A showing the primary vascular parenchyma. Specimen UF 19391-63016 XS peel 10. (E) Tangential longitudinal section through the tall cells of the primary vascular parenchyma (center right), ray cells (center left) and juvenile wood (far left). UF 19391-63016 LS peel 16. (F) Transverse section showing wide solitary vessels (WV) and narrow vessels in long radial multiples (at arrow). Specimen UF 19391-63026 XS peel 6. (G) Tangential longitudinal section (LS) showing coalescent pit apertures on the vessel wall. Specimen UF 19391-63016 LS peel 6. (H) Tangential longitudinal section showing alternate polygonal pits on the vessel wall (at arrow). Specimen UF 19391-63016 LS peel 7. (I) Tangential longitudinal section showing narrow vessels (NV) with oblique end walls, and wide vessels (WV) with simple perforation plates and end walls perpendicular to lateral walls (right arrow). Specimen UF 19391-63026 TLS peel 1. Scale bars: A = 3 mm; B = 1 mm; C, F, I = 200 μm; D, E = 500 μm; G, H = 100 μm.

https://doi.org/10.1371/journal.pone.0248369.g002

Etymology. The genus comes from the Greek *ámpelos*, meaning vine, and *ríza* meaning root. The specific epithet comes from the Greek *héteros* meaning different and *-xylon* meaning wood, referring to the different sizes of the peripheral secondary vascular strands found in Paullinieae.

Description (descriptio generico-specifica). The holotype is an axis 1 cm wide and 3 cm long; the paratype is an axis 0.5 by 1 cm wide and 2.5 cm long. Each consists of bark with one or two peripheral secondary vascular strands (Fig 2A–2C), surrounding a central woody cylinder with a small core of primary vascular parenchyma (Fig 2A–2C). The peripheral vascular strands consist of secondary xylem and phloem derived from C-shaped cambia that lack primary vascular parenchyma. In the holotype, the two preserved peripheral strands are of different sizes. One is c. 3.3 mm by c. 2.0 mm in transverse section and the other is 0.7 mm by c. 0.4 mm (Fig 2A lower arrow, Fig 2C). Primary vascular parenchyma in the central cylinder of the holotype is an eccentric collection of parenchyma cells 200 μm tall by 500 μm wide (Fig 2D). Radial files of cells with dark contents also extend away from the center of the central cylinder

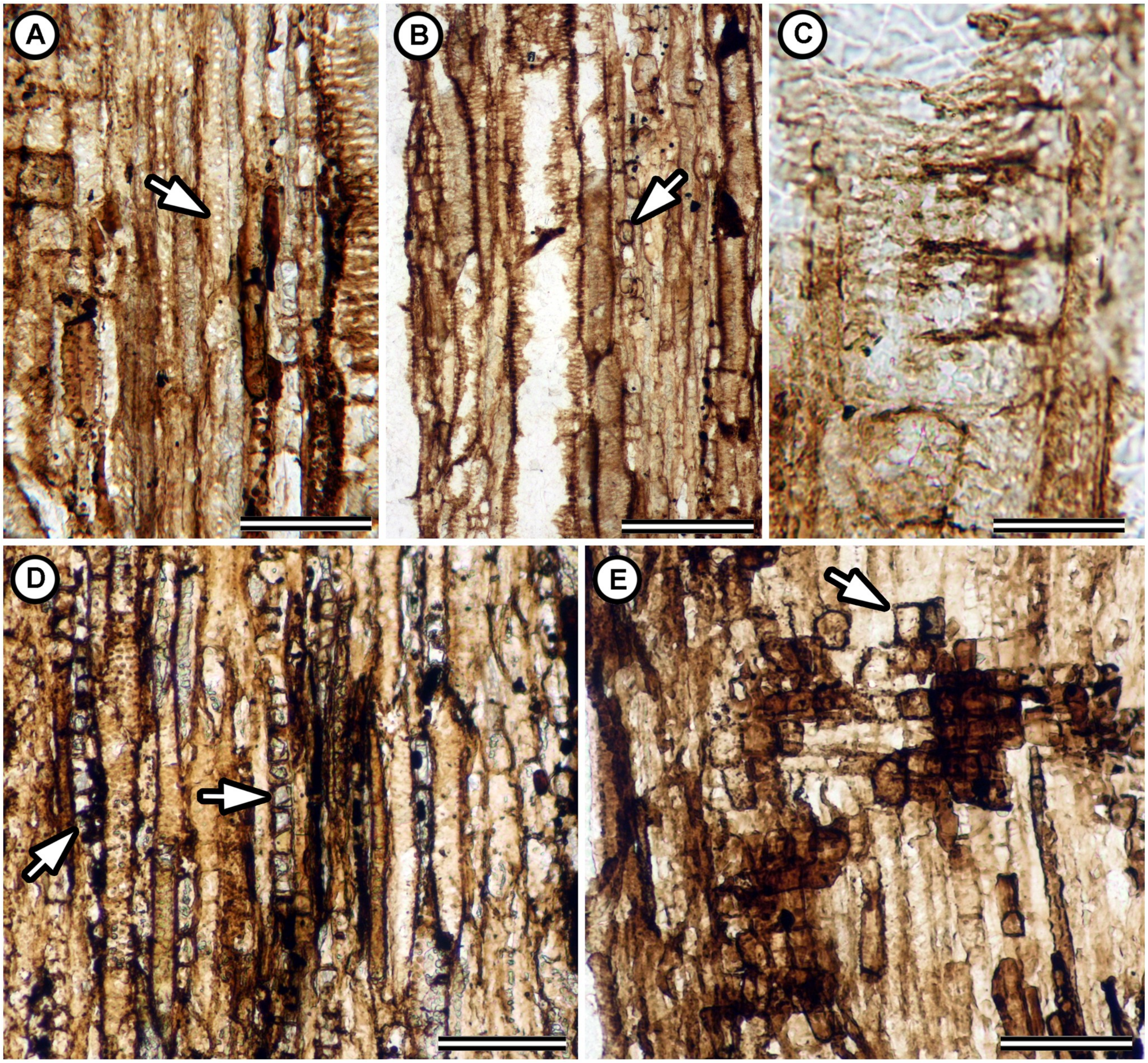

Fig 3. Wood anatomy in *Ampelorhiza heteroxylon*. (A) Tangential longitudinal section showing uniseriate pitting on the fiber walls. Specimen UF 19391-63016 LS peel 5. (B) Tangential longitudinal section showing axial elements including narrow vessels and uniseriate rays (at arrow). Specimen UF 19391-63016 LS peel 1. (C) Radial longitudinal section showing ray cells against a vessel. Note the partially preserved vessel-ray parenchyma pitting similar in size to the intervessel pitting (at arrow). Specimen UF 19391-63016 LS peel 7. (D) Tangential longitudinal section showing uniseriate and biseriate rays (left arrow) and axial elements with crystals (right arrow). Specimen UF 19391-63016 LS peel 5. (E) Radial longitudinal section showing upright (at arrow), square, and procumbent ray cells. Specimen UF 19391-63026 LS peel 2. Scale bars: A = 70 μm; B = 150 μm; C = 40 μm; D, E = 100 μm.

https://doi.org/10.1371/journal.pone.0248369.g003

on one side (Fig 2D). The primary vascular parenchyma cells are tall (c. 150–300 μm), and many have dark contents in the lumen (Fig 2E). Secondary xylem is diffuse porous (Fig 2A & 2F). Growth rings are indistinct (Fig 2A & 2F). Vessels are in two distinct size classes: wide vessels 50-270 μm (mean: 104 μm) in tangential diameter, mostly solitary but also in tangential multiples of 2–3; narrow vessels are 11–50 μm in tangential diameter and arranged in radial multiples of 2–9 (Fig 2A & 2F). Vessel elements are 153–280 μm long (mean: 223 μm, n = 14).

Mean vessel frequency is 27 per mm^2. Vessel element end walls are without scalariform bars; perforation plates are simple (Fig 2I). Tyloses and helical thickenings were not observed. Intervessel pits alternate with distinct borders and coalescent apertures on the walls of large vessels (Fig 2G & 2F). Vessel-ray parenchyma pits were difficult to observe; we did not find large simple pits different from those on the vessel walls (Fig 3C). Fibers are poorly preserved but appear non-septate with minutely bordered uniseriate pits on the radial walls (Fig 3A). Axial parenchyma is diffuse and scanty paratracheal, with strands at least 2–4 cells tall and often chambered with prismatic crystals (Fig 3D). Rays are 1–2 (rarely three) cells wide, less than 1 mm tall, and heterocellular with rows of procumbent square and upright cells mixed throughout (Fig 3E). Secretory structures were not observed.

Remarks. Although cambial variants are often associated with the climbing habit, the presence of peripheral vascular strands is not sufficient to identify the fossils as stems or roots. Bastos et al. [16, 66] demonstrated that cambial variants can be found in both organs. In stems of Paullinieae, the pith is conspicuously angular (e.g., triangular, pentangular) in transverse section with primary vascular bundles at the corners [19]. By contrast, in roots the primary vascular parenchyma is diarch and this region (i.e., the "medulla") is oval and smaller than the stem pith in transverse section (Fig 4). In *Ampelorhiza heteroxylon*, there is an eccentric oval-shaped parenchymatous core c. 200 by 500 μm in diameter (Fig 2D); therefore, our interpretation is that the specimens are roots.

We initially recognized that these fossils might be lianas based on the diameter of the largest vessels relative to the width of the axis. To illustrate this approach, we used logistic regression to classify unknown fossil axes from Lirio East as climbers or self-supporters based on maximum vessel diameter and diameter of the central woody cylinder (S2 Fig). The model was trained using a dataset of 71 samples obtained from Ewers et al. [71], and predicted the habit of 22 fossil axes with woody cylinders greater than 5 mm in diameter from the Lirio East fossil collections. Although the model did predict that the *Ampelorhiza* fossils (and the *Rourea* fossil described by Jud and Nelson [37]) are climbers, the training dataset is only stem material and therefore may not be suitable for classifying roots, given the patterns found by Ewers et al. [72] when comparing stems and roots in lianas and trees. Further work on the relationship between hydraulic path length, vessel diameter, and root diameter in lianas and self-supporters (as has been done for stems [73]) would be useful for identifying lianas in the fossil record.

Fossil leaves

We found one fossil species assigned to *Serjania* and two assigned to *Cardiospermum* from North America in the literature (Table 1). All three were described from fossils of isolated leaflets or partially complete compound leaves (Fig 5). MacGinitie [74] described *"Serjania" rara* based on leaves from the Eocene Aycross Formation in northwestern Wyoming. The same species also occurs in the Eocene Bridger Formation in southwestern Wyoming [75]. *"Cardiospermum" terminale* (Lesquereux) MacGinitie was first described from the Eocene Florissant Formation in central Colorado by Lesquereux [76] as *Lomatia*. MacGinitie [77] transferred these specimens and others to *Cardiospermum* based on the twice-ternate leaf organization and architecture of lobes, teeth, and major vein framework of the leaflets. This species was later reported from the late Eocene to early Oligocene Climbing Arrow Member of the Renova Formation in southwestern Montana [78, 79] as well. Finally, *"Cardiospermum" coloradensis* (Knowlton) MacGinitie was first described from the Eocene Green River Formation as *Phyllites* by Knowlton [80]; and later transferred to *Cardiospermum* by MacGinitie [81]. This species has been reported from throughout the Green River Formation [81–84]. Updated descriptions of these three species are provided in the (S2 Appendix).

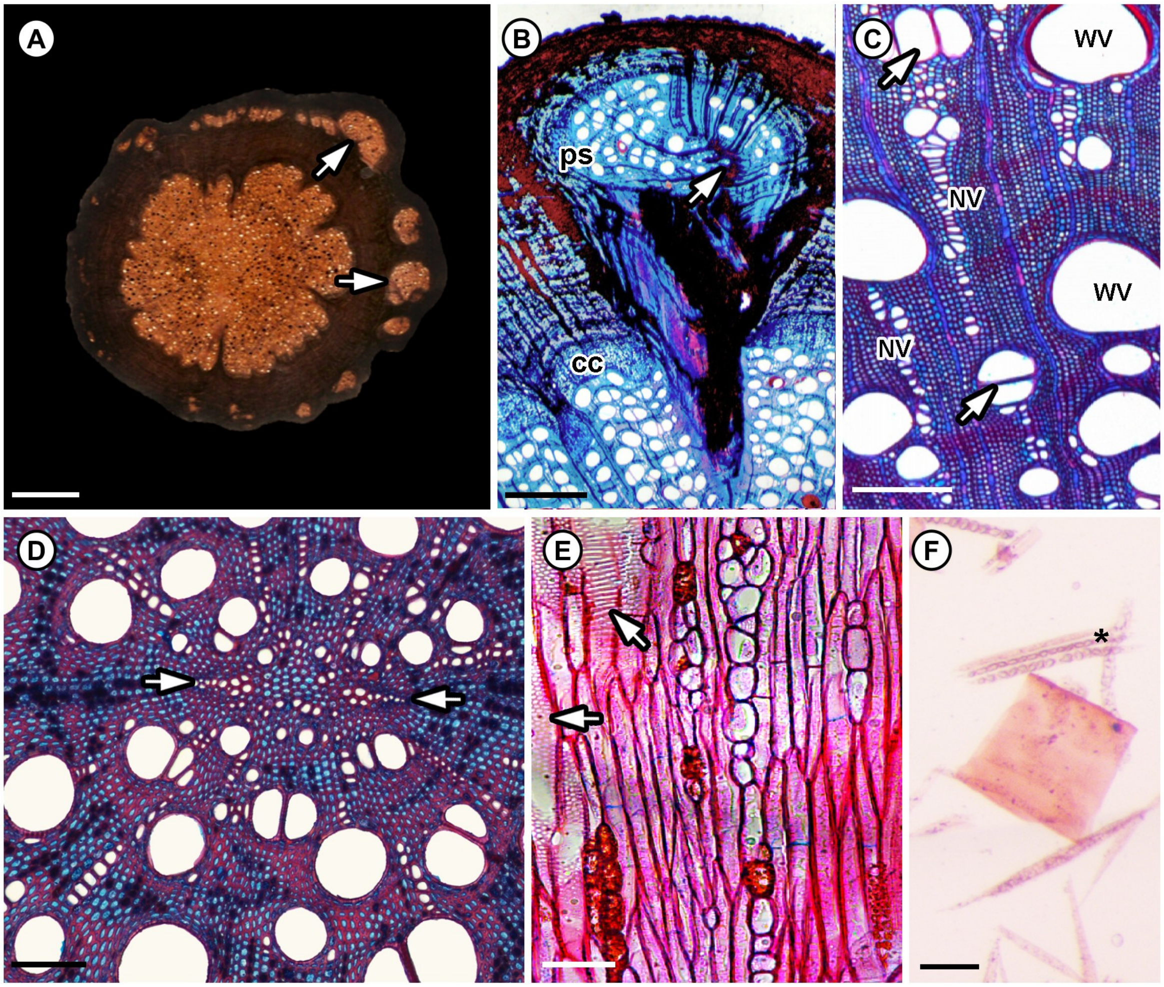

Fig 4. Wood anatomy of the roots of extant Paullinieae species. A–B: Neoformations forming peripheral vascular strands in *Serjania caracasana* (Jacq.) Willd. in transverse section. (A) Root macromorphology presenting a cambial variant. Arrows point to individual peripheral vascular strands. (B) Close up of the juncture of the central cylinder (cc) and a peripheral vascular strand (ps) with a c-shaped "pith" (i.e., primary vascular parenchyma of the root). (C) Secondary xylem of *Thinouia scandens* Triana & Planch. with vessel dimorphism in transverse section. Note the wide vessels (WV) are solitary or in tangential (upper arrow) or radial multiples (lower arrow), while the narrow vessels (NV) are in longer radial chains. (D) Primary vascular parenchyma in the center of the the diarch roots (arrows towards protoxylem poles) of *S. caracasana* in transverse section. (E) Alternate intervessel pits (lower arrow) and those with coalescent apertures (upper arrow) in *S. caracasana* in tangential longitudinal section. (F) Prismatic crystals in the axial parenchyma (*) of *S. caracasana* in macerated material. Scale bars: A = 0.5 cm, B = 1 mm, C = 250 μm, D = 100 μm, E = 70 μm, F = 50 μm. *prismatic crystals in axial parenchyma.

https://doi.org/10.1371/journal.pone.0248369.g004

The extinct genus *Bohlenia* Wolfe & Wehr [85] was established for sapindaceous leaves and fruits from the Eocene Republic flora (Klondike Mountain Formation) in Washington, USA (Table 1). Wolfe and Wehr [85] suggested that *B. americana* (Brown) Wolfe & Wehr may belong to Paullinieae based on the course of the secondary veins and on the assumption that co-occurring samaras belonged to the same species; however, McClain and Manchester [86] transferred the samaras to *Dipteronia brownii* McClain & Manchester and noted that *Bohlenia* foliage is similar to *Koelreuteria elegans* (Seem.) A.C. Sm. Both of these fossil species are members of Sapindaceae, but neither belong to Paullinieae.

We also found three species assigned to *Serjania* from the Cenozoic of Brazil in the literature (Table 1). Fittipaldi et al. [87] described *Serjania lanceolata* Fittipaldi, Simões Giulietti et Pirani and *Serjania itaquaquecetubensis* Fittipaldi, Simões Giulietti et Pirani based on

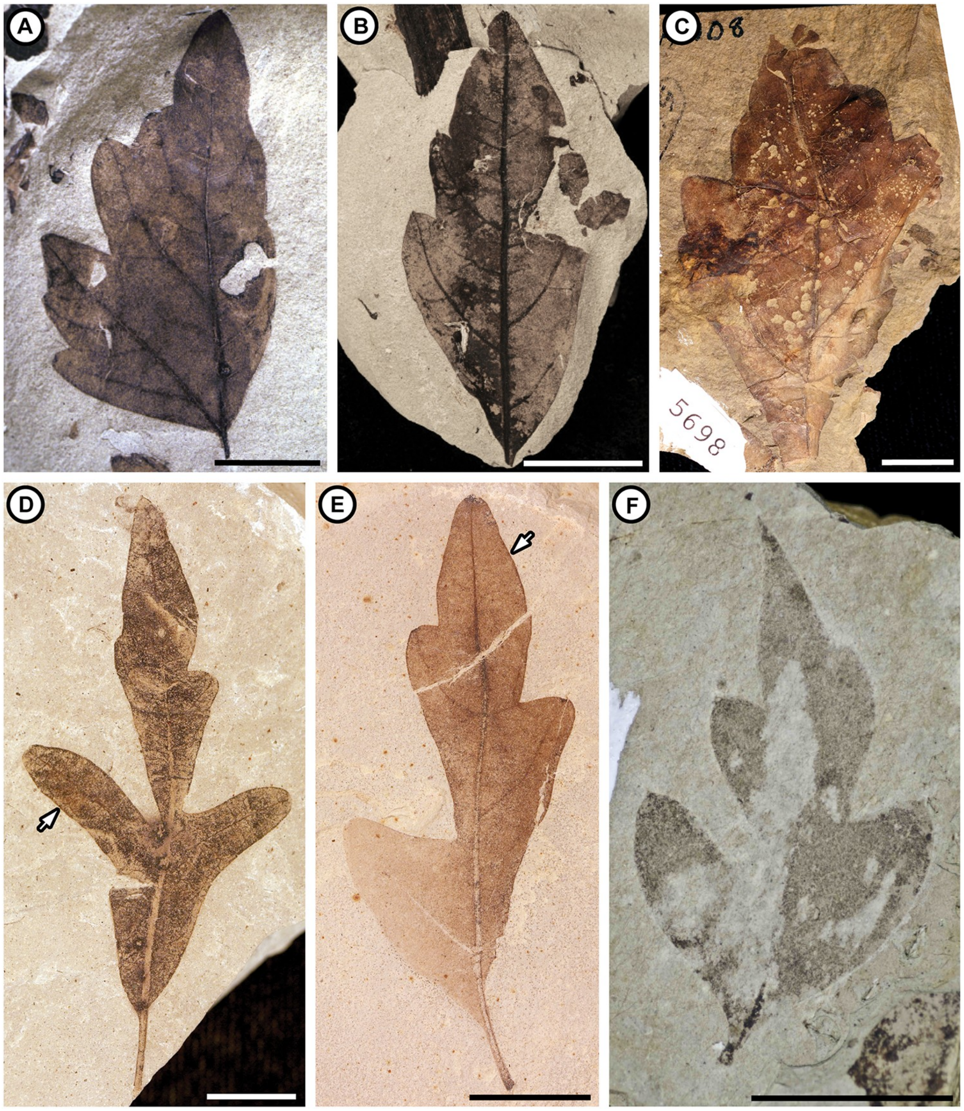

Fig 5. Leaf fossils previously assigned to Paullinieae. (A) *"Serjania" rara* MacGinitie from the Bridger Formation, Blue Rim site, Sweetwater County, Wyoming, UF 15761S-57786. (B) *"Serjania" rara* MacGinitie from the Bridger Formation, Blue Rim site, Sweetwater County, Wyoming, UF 15761N-61430. (C) Paratype of *"Serjania" rara* MacGinitie from the Aycross Formation, Kisinger Lakes site, northwestern Wyoming (Pl 25, Fig 3 in [74]), UCMP PA 108, 5698. (D) Hypotype of *"Cardiospermum" coloradensis* (Knowlton) MacGinitie from the Green River Formation, west of Wardell Ranch site, Colorado (Pl 22, Fig 3 in [81]), UCMP PA 321, 20593. Arrow indicates marginal vein. (E) *"Cardiospermum" coloradensis* (Knowlton) MacGinitie from the Green River Formation in Rainbow, UT, UCMP PB02016, 201265. Arrow indicates marginal vein. (F) *"Cardiospermum" terminale* (Lesquereux) MacGinitie from the Florissant Formation in central Colorado, FLFO 10147. Scale bars = 1 cm.

https://doi.org/10.1371/journal.pone.0248369.g005

incomplete unlobed, toothed leaf blades from the Oligocene upper Itaquaquecetuba Formation. To our knowledge, the characteristic pollen of Paullinieae has not been recognized in palynological studies of this formation [88]. Finally, *Serjania mezzalire* Duarte et Rezende-Martins was described from fossil leaves in the Miocene Rio Claro Formation [89, 90].

Edwards and Wannacot [91] compiled list of all fossil species that had been assigned to *Paullinia* based on leaf morphology from Europe. They concluded that a close relationship to extant Paullinieae can be rejected or is doubtful for all of them based on morphology or quality of preservation. We concur, so we did not consider these further.

There is considerable variation in the blade shape, margin type, tertiary venation, and base shape among extant Paullinieae (Fig 6). Leaf margins may be unlobed or lobed, toothed or untoothed. Toothed margins may be serrate, dentate, or crenate. Secondary vein framework may be craspedodromous, semicraspedodromous, brochidodromous, or eucamptodromous. Leaf organization is also variable. Leaves may be simple, once or twice imparipinnate, or up to thrice ternate (most commonly twice ternate). In compound leaves, the rachis may be winged or unwinged. Axillary tendrils may be absent or present. Many of these characters also vary across Sapindaceae. Based on our observations, isolated fossil leaves or leaflets of Paullinieae may be recognizable if they preserve a combination of the following characters: Axial tendrils, stipules, ternate compound organization, winged rachides, and absence of a marginal vein.

Morphological similarities between *"Cardiospermum" coloradensis*, *"C." terminale*, *"Serjania" rara*, and the leaves of some extant Paullinieae include 1. ternate-compound organization, 2. decurrent (Figs 5C, 5E and 5F and 6A and 6B) or complex leaflet bases (Fig 5A and 5B), 3. irregular spacing of secondary veins, 4. secondary veins that terminate beyond the apex of lobes/teeth, 5. secondary veins that terminate in angular ("V-shaped") sinuses (Fig 5), and 6. secondary veins that bifurcate around angular sinuses (Fig 5E). However, some or all of these characters can be found in the leaves of other Sapindaceae (e.g., *Thouinia* Poit., *Koelreuteria* Laxm., *Dipterodendron* Radlk., *Dilodendron* Radlk., and *Athyana* (Griseb.) Radlk) and in other families (see Discussion section for further commentary); they are not diagnostic of *Cardiospermum*, *Serjania*, nor Paullinieae. Furthermore, a prominent marginal vein like that present in at least some specimens of the fossil species (Fig 5D and 5E) is not present in extant *Serjania* and *Cardiospermum* (Fig 6A and 6B). The descriptions and images of *"Serjania" lanceolata*, *"S." itaquaquecetubensis*, and *"S." messalire* show the shape of the blade, the presence of a serrate margin, and craspedodromous secondary vein framework [87, 90]. Although these characters are consistent with *Serjania*, their combination is not diagnostic of the genus.

Phylogenetic position of *Ampelorhiza*

We evaluated the placement of *Ampelorhiza* by observation of the allcompat consensus, MCC, and MAP trees sampled from the posterior distribution. *Ampelorhiza* is always nested within extant Paullinieae, however its relationship with extant genera differs based on the method used to generate the tree, reflecting the uncertainty typical of taxa with a high proportion of missing data. In the allcompat consensus tree (Fig 7) *Ampelorhiza* is nested within a clade with *Cardiospermum*, *Paullinia*, and *Serjania*. The various positions of *Ampelorhiza* within this clade is represented as a polytomy that includes several lineages of *Serjania* and *Cardiospermum*. In the maximum *a posteriori* tree (S1 Appendix), *Ampelorhiza* is nested within *Urvillea*, whereas in the maximum clade credibility tree *Ampelorhiza* is nested within *Serjania*. These results further supports our circumscription of *Ampelorhiza* as a distinct genus from extant Paullinieae. The placement of *Ampelorhiza* within Paullinieae is supported by vessel dimorphism, heterocellular rays, and axial parenchyma strands typically 2-4 cells long. One synapomorphy of Paullinieae that we did not observe in the fossil is wide rays (ray dimorphism); however, we only examined two root fragments and this character is observed in many, but not all, samples from modern roots [16].

Discussion

Roots

The combination of peripheral vascular strands (Figs 2A–2C and 4A & 4B), vessel dimorphism (Figs 2F & 2I and 4B–4D), wide vessels solitary or in tangential multiples of 2–3 (Fig 2F and 4C), narrow vessels in long radial multiples of 2–21 (Figs 2F and 4C & 4D), alternate

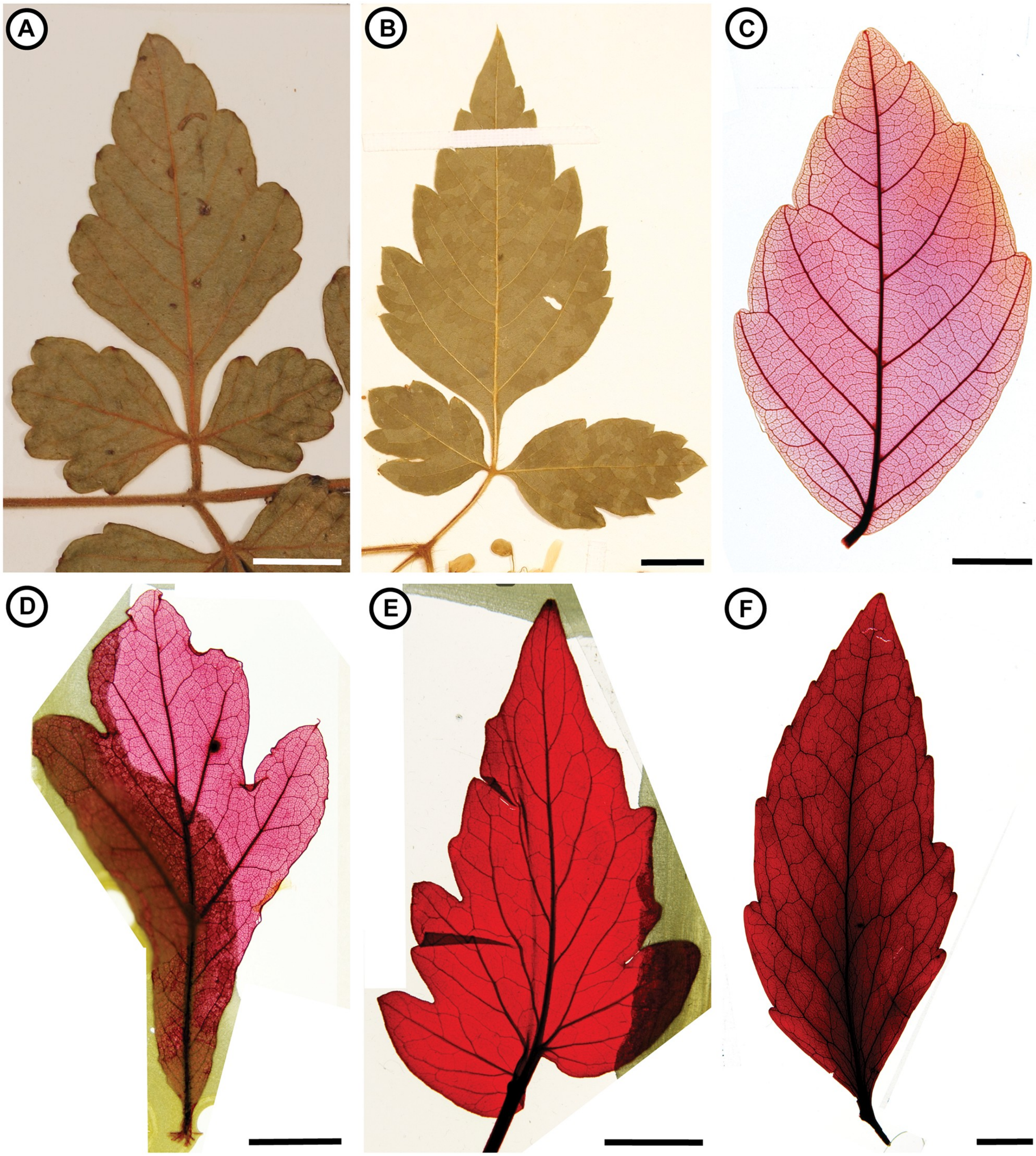

Fig 6. Extant leaves. Modern leaves for comparison with the putative Paullinieae fossils. Cleared leaves from the National Cleared Leaf Collection (NCLC). (A) *Serjania rhombea* Radlk. (Coll.: W.H. Lewis, J.D. Dwyer, T.S. Elias, and R. Solís #72 (UC 1355158), 1966, Panama]. (B) *Cardiospermum halicacabum* L. [Coll.: R.D.A. Baylis #5080 (UC 1409568), 1972, South Africa]. (C) *Paullinia pinnata* L., NCLC 0012. (D) *Quercus nigra* L., NCLC 0215. (E) *Lycopersicum esculentum* L., NCLC 1640. (F) *Beauprea balansae* Brongn. & Gris, NCLC 6658. Scale bars = 1 cm.

https://doi.org/10.1371/journal.pone.0248369.g006

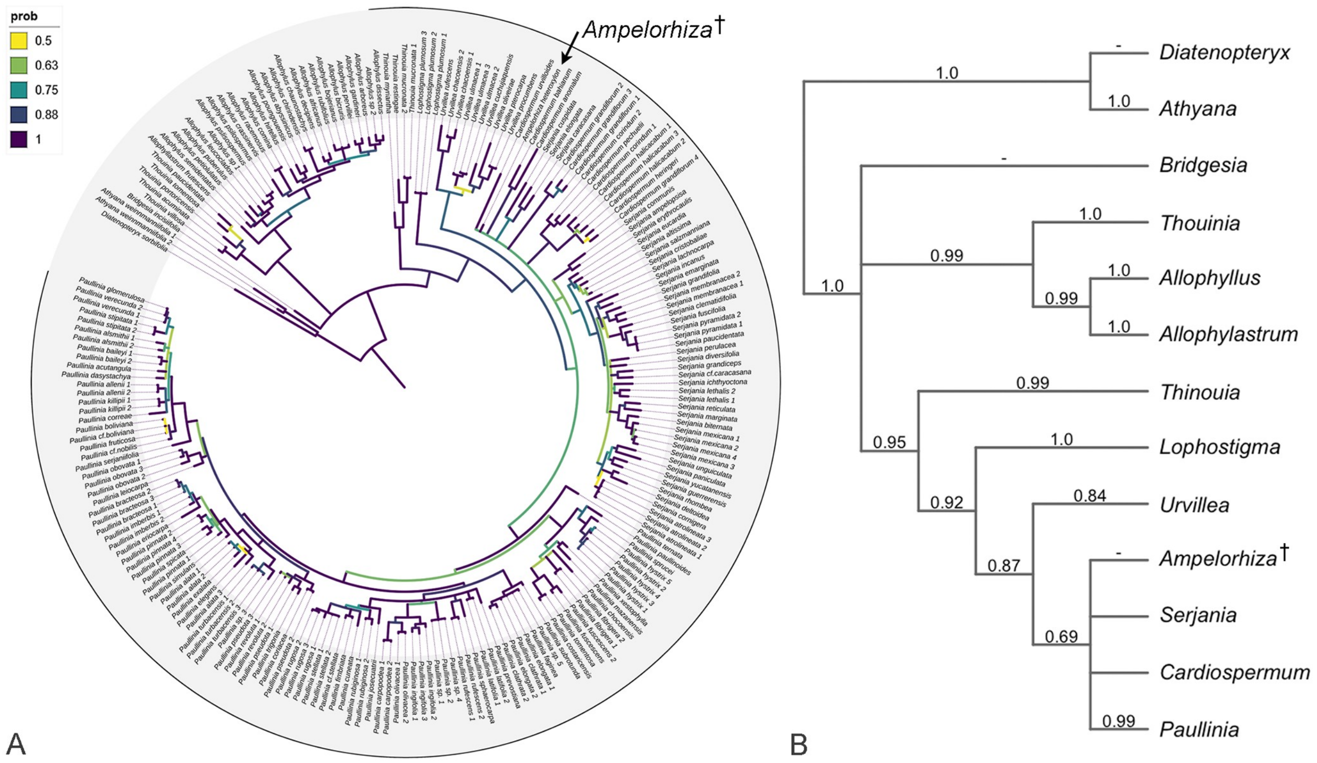

Fig 7. Phylogeny of supertribe Paulliniodae. (A) Majority rule consensus tree with all compatible groups ("allcompat") of supertribe Paulliniodae sensu Acevedo et al. [21], generated in MrBayes 3.2.7 from an anatomical and molecular combined dataset of 216 tips. Branch colors indicate posterior probabilities. The outermost black line indicates the tribe Paullinieae. Note the position of the fossil taxon *Ampelorhiza* within Paullinieae indicated by the arrow and the dagger. (B) Summary tree showing the same topology, but pruned to show genera only, assuming all genera are monophyletic. Numbers above branches are posterior probabilities, dashes indicate genera represented by a single species in the "allcompat" consensus tree.

https://doi.org/10.1371/journal.pone.0248369.g007

intervessel pits with slit-like coalescent apertures (Figs 2G and 2H and 4E), heterocellular rays, prismatic crystals in axial parenchyma (Figs 3D and 4F), and dark content (possibly phenolic compounds) in primary vascular parenchyma and ray parenchyma (Fig 2D and 2E) support the inclusion of *Ampelorhiza* in Paullinieae [13, 16, 18, 64, 66, 92, 93, 94]. Two wood anatomical characters typical of extant Paullinieae were not observed in the fossils: 1) alternating bands of thin and thick-walled regions in the wood which may either be axial parenchyma alternating with ordinary fibers (e.g., *Serjania* spp.) or parenchyma-like fiber bands alternating with ordinary fibers (e.g., *Paullinia* spp.) and 2) ray dimorphism. Because the bands are clearest in sufficiently thin, stained sections or polished blocks, it may be that the thickness of the peels and the absence of stain obscures this feature.

The cambial configuration in stems and roots is highly variable in Paullinieae. Chery et al. [19] and Cunha Neto et al. [18] together distinguished six ontogenetic pathways in the stems of *Paullinia* alone, and we expect that *Serjania* has the most variation in the tribe based on preliminary observations of images in the Smithsonian Liana databases (Acevedo & Chery, personal observation). Furthermore, Bastos [16, 66] showed that roots may or may not also have cambial variants, and when present they do not necessary mirror the configuration of the stems. An asymmetrical distribution of peripheral secondary vascular strands of different sizes, as in *Ampelorhiza heteroxylon*, occurs in the roots of *Serjania caracasana* (Fig 4A & 4B) and the stems of some *Paullinia* [18]. Given the variation among stems and the paucity of data on cambial variants in roots, the configuration of secondary growth in the fossils does not justify assignment to one of the extant genera.

Despite some anatomical differences among the genera of Paullinieae, the fossils of *Ampelorhiza* do not preserve a combination of wood anatomy characters diagnostic of any extant genus either, they are most similar to some *Serjania*. The wood of *Serjania* stems has banded axial parenchyma, no septate fibers, and crystals confined to axial elements, whereas *Paullinia*, *Thinouia*, and *Cardiospermum* have scanty axial parenchyma, abundant septate fibers, and crystals in ray parenchyma. *Thinouia* differs from *Paullinia* and *Cardiospermum* by the absence of crystals in axial elements [13], and some *Paullinia* can be recognized by a herringbone pattern in the wide rays when viewed in transverse section [13]. The fossils do not have banded parenchyma, nor do they have wide rays with a herringbone pattern. They do have crystals in the axial elements but we did not observe them in the rays, nor did we detect septate fibers.

Leaves

We reject the generic assignments of *Cardiospermum* and *Serjania* species described from fossil leaf material. Our search for leaves with organization, margin features, and venation architecture similar to *"C." coloradensis*, *"C." terminale*, and *"S." rara* outside of Sapindaceae led to comparisons with Anacardiaceae (e.g., *Rhus* L.), Fagaceae (e.g., *Quercus* L.), Proteaceae (e.g., *Roupala* Aubl., *Lomatia* R. Br., *Beauprea* Brongn. & Gris), Ranunculaceae (e.g., *Clematis* L.), and Solanaceae (e.g., *Hyoscyamus* L., *Chamaesaracha* (A. Gray) Benth. & Hook. f., *Physalis* L., *Lycopersicum* Hill.). Some *Rhus* (Anacardiaceae) have similar shapes to the fossil material, but secondary venation in *Rhus* varies from craspedodromous to cladodromous. Some Fagaceae have similar blade shape, secondary veins, and major veins that project beyond the margin of the blade; however, all Fagaceae have simple leaves and the sinuses are generally rounded rather than angular as in the fossils. Previous authors (e.g., [76, 83]) have attributed fossils like these to Proteaceae; however, although secondary veins in the Proteaceae are variable (e.g., brochidodromous to semicraspedodromous to festooned brochidodromous to festooned semicraspedodromous), they are unlike the craspedodromous framework in the fossils and again the sinuses between teeth are generally rounded in Proteaceae rather than angular. The compound leaves of some lobed and toothed *Clematis* (Ranunculaceae) can be distinguished from the fossils because they usually have festooned secondary venation. Finally, several Solanaceae have asymmetric blades and similarly shaped teeth and lobes; however, again the sinuses tended to be rounded rather than angular as in the fossils.

Leaf architectural characters preserved in *"C." coloradensis*, *"C." terminale*, and *"S." rara* support inclusion in Sapindaceae, yet we consider a close relationship with Paullinieae unlikely based on the presence of a prominent perimarginal vein in the fossils and the absence of co-occurring fossil fruits or pollen despite decades of intensive sampling in the Green River Formation and the Florissant fossil beds. Similarly, in his update of the fossil flora of Florissant, Manchester [95] doubted the generic assignment of *"C." terminale* based on the rather coriaceous texture of the fossils compared to extant *Cardiospermum* and the absence of associated fruits. Other extant Sapindaceae with similar leaf organization, margin type, teeth, and venation include: *Thouinia* Poit., *Koelreuteria* Laxm., *Dipterodendron* Radlk., *Dilodendron* Radlk., and *Athyana* (Griseb.) Radlk.

Evolution of Paullinieae

To our knowledge, the oldest reliable fossil evidence of Paullinieae is heteropolar hemi-tri-syncolpate pollen from the Gatuncillo Formation in Panama [52]. Some fossil species of the genera *Syncolporites* and *Proteacidites* (used for dispersed pollen) may belong to Paullinieae (or Proteaceae or Myrtaceae) [96]; however, a review of those species is beyond the scope of this

work. Heteropolar hemi-tri-syncolpate pollen is a synapomorphy of the clade that includes all Paullinieae except *Thinouia* and *Lophostigma* [21, 97, 98]. Therefore, these fossils can be considered evidence of crown-group Paullinieae in the fossil record. Unfortunately, constraining the age of these samples is challenging. Montes et al. [99] reported Late Eocene and Oligocene foraminifera from the Gatuncillo Formation, consistent with the original age estimate from Graham [52]. More recently, Ramírez et al. [100] obtained detrital zircons from two sites that constrain the maximum age of deposition of the Gantuncillo Formation to Late Eocene, c. 41 Ma and c. 36 Ma respectively, but we do not know their position relative to Graham's [52] pollen sample. Older putative occurrences of Middle Eocene pollen from the Wagon Bed Formation in Wyoming [101] and the Claiborne Group in northern Alabama [102] were not described nor figured, and are not reliable [103]. Pollen from the Kisinger Lakes paleoflora in Wyoming that MacGinitie compared with *Serjania* [74] was not described; however, one figure shows a single grain 24 μm across in polar view with a 3-(parasyncol)porate structure. It is not possible to determine whether it was heteropolar and pollen grains in Paullinieae are larger than 30 μm across [98, 104]. Therefore, we do not consider this a reliable fossil occurrence of Paullinieae based on the available information. Younger occurrences include heteropolar demisyncolpate pollen from the late Miocene Gatun Formation in Panama [43, 49] and the Pliocene Paraje Solo Formation, also in Panama [47].

The transition to the liana habit occurred only once in Sapindaceae along the branch leading to crown-group Paullinieae [21]. Accordingly, all members of the tribe share anatomy associated with the climbing habit such as abrupt changes in vessel diameter, vessel dimorphism, and numerous members have cambial variants [19, 105]. The combination of wood anatomical characters and the presence of the peripheral vascular strands preserved in the fossils provides strong evidence of the climbing habit in Paullinieae by the early Miocene.

Paleoecology

Lianas are a conspicuous element of tropical forests and their fossils contribute to reconstructions of paleoenvironments and paleocommunities. The Lirio East fossil assemblage includes at least 32 plant morphotypes have been distinguished and assigned to family based on fossil fruits, seeds, and woods [32–34, 36–38]. The discovery of *Ampelorhiza* brings the number of liana species to a minimum of 8, or 25% of the local assemblage. This value is typical of lowland tropical forests [106]. Three other potential liana axes were identified using logistic regression (S2 Fig), but remain to be described (F. Herrera, pers. comm.). At least 31 additional fruit and seed morphotypes have been distinguished but not yet identified to family [32]. In modern tropical forests liana species richness is highest in seasonally dry tropical forests and locally near forest edges or in treefall gaps [107–109]. Given the rarity of distinct growth rings in the co-occurring fossil woods and the preference of *Sacoglottis* and *Oreomunnea* for humid tropical forests [33, 34], we hypothesize that the high proportion of lianas in the Lirio East assemblage is a signal of riparian zone disturbance and/or edge effects in a humid tropical forest on a landscape shaped by nearby volcanic activity [31].

Conclusion

The discovery of *Ampelorhiza* reported here is the oldest reliable macrofossil evidence of Paullinieae. Fossil leaves from the Eocene of North America previously attributed to *Cardiospermum* and *Serjania* likely belong to Sapindaceae, but are not reliable occurrences of Paullinieae. Our findings support the conclusion that diversification of the tribe was underway by at least 18.5–19 Ma (early Miocene) and that the climbing habit had evolved by that time.

Supporting information

S1 Appendix. Folder containing the accession list, mrbayes infile.nex, mcc, map, allcompat, and accession list.
(ZIP)

S2 Appendix. Revised descriptions of the leaf architecture. Descriptions of *Bohlenia americana*, *Bohlenia insignis*, *"Cardiospermum" coloradensis*, *"Cardiospermum" terminale*, and *"Serjania" rara*.
(PDF)

S1 Fig. Transverse section of the paratype, UF 19391-63026.
(TIF)

S2 Fig. Plot of lianas and self-supporting woody dicots. Filled points are fossil axes from the Lirio East site classified as either climbers or self-supporters using logistic regression. We applied a conservative decision threshold of 0.95 for classifying lianas.
(TIF)

Acknowledgments

We thank Bruce MacFadden, Jonathan Bloch, Steven Manchester, Carlos Jaramillo, and Fabiany Herrera for support in the early phases of this project, Veronica Angylossy for supervision over Carolina Basto's thesis work concerning the root anatomy of Paullinieae, and Lillian Pearson for making initial peels of the fossil during her PCP-PIRE internship. Fabiany Herrera discovered the fossil site at Lirio East. We thank Ricardo Martinez for donating the vehicles used for fieldwork in Panama, and the Autoridad del Canal de Panama (ACP) for access to the site where the fossils were collected. We also thank the staff of the herbaria at the University of California Berkeley, the Florida Museum of Natural History, and the University of Kansas, and the staff of the paleobotany collections at UC Berkeley, the Florida Museum of Natural History, and the Smithsonian for assistance. We thank Sarah DeWitt for comments on the figures. Finally, we thank the reviewers for helpful feedback and suggestions during the review process. Any opinions, findings, conclusions, or recommendations expressed in this article are those of the authors and do not necessarily reflect the views of the NSF.

Author Contributions

Conceptualization: Nathan A. Jud, Joyce G. Chery.

Data curation: Nathan A. Jud, Joyce G. Chery.

Formal analysis: Nathan A. Jud, Joyce G. Chery.

Investigation: Nathan A. Jud, Sarah E. Allen, Chris W. Nelson, Carolina L. Bastos, Joyce G. Chery.

Methodology: Nathan A. Jud, Joyce G. Chery.

Resources: Carolina L. Bastos.

Supervision: Nathan A. Jud.

Writing – original draft: Nathan A. Jud, Sarah E. Allen, Chris W. Nelson, Joyce G. Chery.

Writing – review & editing: Nathan A. Jud, Sarah E. Allen, Joyce G. Chery.

References

1. Acevedo-Rodríguez P, Van Welzen PC, Adema F, Van Der Ham RWJM. Flowering Plants. Eudicots. Sapindaceae. Springer p. 357–407. 2011.
2. Harrington MG, Edwards KJ, Johnson SA, Chase MW, Gadek PA. Phylogenetic Inference in Sapindaceae sensu lato Using Plastid matK and rbcL DNA Sequences. Systematic Botany. 2005; 30(2):366–382. https://doi.org/10.1600/0363644054223549
3. Buerki S, Forest F, Acevedo-Rodríguez P, Callmander MW, Nylander JAA, Harrington M, et al. Plastid and nuclear DNA markers reveal intricate relationships at subfamilial and tribal levels in the soapberry family (Sapindaceae). Molecular Phylogenetics and Evolution. 2009; 51(2):238–258. https://doi.org/10.1016/j.ympev.2009.01.012 PMID: 19405193
4. Buerki S, Lowry PP II, Alvarez N, Razafimandimbison SG, Küpfer P, Callmander MW. Phylogeny and circumscription of Sapindaceae revisited: molecular sequence data, morphology and biogeography support recognition of a new family, Xanthoceraceae. Plant Ecology and Evolution. 2010; 143(2):148–159. https://doi.org/10.5091/plecevo.2010.437
5. Tamaio N, Somner GV. Development of corded vascular cylinder in *Thinouia restingae* Ferruci & Somner (Sapindaceae: Paullinieae). The Journal of the Torrey Botanical Society. 2010; 137(4):319–326. https://doi.org/10.3159/10-RA-047.1
6. Schenck H. Beiträge zur Biologie und Anatomie der Lianen im besonderen der in Brasilien einheimischen Arten. 4-5. G. Fischer; 1892. f
7. Radlkofer L. Sapindaceae In: Engler A. & Prantl K. Die Natürlichen Pflazenfamilien. 1895; 3:277–366.
8. Pfeiffer HH. Das abnorme Dickenwachstum. vol. 9. Gebr. Borntraeger; 1926.
9. Radlkofer L, Engler A, Diels L. Sapindaceae. Engelmann 1933.
10. Johnson MA, Truscott FH. On the Anatomy of *Serjania*. I. Path of the Bundles. American Journal of Botany. 1956; 43(7):509–519. https://doi.org/10.1002/j.1537-2197.1956.tb10525.x
11. Obaton M. Les Lianes ligneuses a structure anormale des forêts denses d'Afrique occidentale. Annales de scinences naturelles (Botanique) Ser. 12. 1960; 1:1–220
12. Van der Walt JJA, Van der Schijff HP, Schweickerdt HG. Anomalous secondary growth in the stem of lianas *Mikania cordata* (Burm. F.) Robins (Compositae) and *Paullinia pinnata* Linn.(Sapindaceae). Kirkia. 1973; 9:109–138.
13. Klaassen R. Wood Anatomy of the Sapindaceae. IAWA J. suppl 2. International Association of Wood Anatomists, Leiden; 1999.
14. Tamaio N, Neves MF, Brandes AFN, Vieira RC. Quantitative analyses establish the central vascular cylinder as the standard for wood-anatomy studies in lianas having compound stems (Paullinieae: Sapindaceae). Flora—Morphology, Distribution, Functional Ecology of Plants. 2011; 206(11):987–996. https://doi.org/10.1016/j.flora.2011.07.006
15. Tamaio N. Wood anatomy of lianas of Sapindaceae commercially used in São Paulo-SP. Cerne. 2011; 17(4):533–540.
16. Bastos CL, Tamaio N, Angyalossy V. Unravelling roots of lianas: a case study in Sapindaceae. Annals of Botany. 2016; 118(4):733–746. https://doi.org/10.1093/aob/mcw091
17. Lopes WAL, De Souza LA, De Almeida OJG. Procambial and cambial variants in *Serjania* and *Urvillea* species (Sapindaceae: Paullinieae). Journal of the Botanical Research Institute of Texas. 2017; 11 (2):421–432.
18. Cunha Neto ILD, Martins FM, Somner GV, Tamaio N. Successive cambia in liana stems of Paullinieae and their evolutionary significance in Sapindaceae. Botanical Journal of the Linnean Society. 2018; 186(1):66–88. https://doi.org/10.1093/botlinnean/box080
19. Chery JG, Pace MR, Acevedo-Rodríguez P, Specht CD, Rothfels CJ. Modifications during early plant development promote the evolution of nature's most complex woods. Current Biology. 2020; 30 (2):237–244. https://doi.org/10.1016/j.cub.2019.11.003
20. Acevedo-Rodriguez P. A revision of *Lophostigma* (Sapindaceae). Systematic Botany. 1993; 18 (3):379–388. https://doi.org/10.2307/2419414
21. Acevedo-Rodríguez P, Wurdack KJ, Ferrucci MS, Johnson G, Dias P, Coelho RG, et al. Generic relationships and classification of tribe Paullinieae (Sapindaceae) with a new concept of supertribe Paulliniodae. Systematic Botany. 2017; 42(1):96–114. https://doi.org/10.1600/036364417X694926
22. Chery JG, Acevedo-Rodríguez P, Rothfels CJ, Specht CD. Phylogeny of *Paullinia* L. (Paullinieae: Sapindaceae), a diverse genus of lianas with dynamic fruit evolution. Molecular Phylogenetics and Evolution. 2019; 140:106577. https://doi.org/10.1016/j.ympev.2019.106577
23. Buerki S, Forest F, Alvarez N, Nylander JAA, Arrigo N, Sanmartín I. An evaluation of new parsimony-based versus parametric inference methods in biogeography: a case study using the globally

distributed plant family Sapindaceae. Journal of Biogeography. 2011; 38(3):531–550. https://doi.org/10.1111/j.1365-2699.2010.02432.x

24. Buerki S, Forest F, Stadler T, Alvarez N. The abrupt climate change at the Eocene–Oligocene boundary and the emergence of South-East Asia triggered the spread of sapindaceous lineages. Annals of Botany. 2013; 112(1):151–160. https://doi.org/10.1093/aob/mct106

25. Muellner-Riehl AN, Weeks A, Clayton JW, Buerki S, Nauheimer L, Chiang YC, et al. Molecular phylogenetics and molecular clock dating of Sapindales based on plastid rbcL, atpB and trnL-trnF DNA sequences. Taxon. 2016; 65(5):1019–1036. https://doi.org/10.12705/655.5

26. Erwin DM, Stockey RA. Sapindaceous flowers from the Middle Eocene Princeton chert (Allenby Formation) of British Columbia, Canada. Canadian Journal of Botany. 1990; 68(9):2025–2034. https://doi.org/10.1139/b90-265

27. Gandolfo MA, Nixon KC, Crepet WL. Selection of fossils for calibration of molecular dating models. Annals of the Missouri Botanical Garden. 2008; 95(1):34–42. https://doi.org/10.3417/2007064

28. Retallack GJ, Kirby MX. Middle Miocene global change and paleogeography of Panama. Palaios. 2007; 22(6):667–679. https://doi.org/10.2110/palo.2006.p06-130r

29. Kirby MX, Jones DS, MacFadden BJ. Lower Miocene stratigraphy along the Panama Canal and its bearing on the Central American Peninsula PLoS One. 2008; 3(7):e2791 https://doi.org/10.1371/journal.pone.0002791 PMID: 18665219

30. MacFadden BJ, Bloch JI, Evans H, Foster DA, Morgan GS, Rincon A, et al. Temporal calibration and biochronology of the Centenario Fauna, early Miocene of Panama. The Journal of Geology. 2014; 122 (2):113–135. https://doi.org/10.1086/675244

31. Buchs DM, Irving D, Coombs H, Miranda R, Wang J, Coronado M, et al. Volcanic contribution to emergence of Central Panama in the Early Miocene. Scientific reports. 2019; 9(1):1–16. https://doi.org/10.1038/s41598-018-37790-2 PMID: 30723222

32. Herrera F. Revealing the floristic and biogeographic composition of Paleocene to Miocene Neotropical forests. [PhD Thesis] University of Florida, Gainesville, FL; 2014.

33. Herrera F, Manchester SR, Jaramillo CA, MacFadden BJ, da Silva-Caminha SA. Phytogeographic history and phylogeny of the Humiriaceae. International Journal of Plant Sciences. 2010; 171(4):392–408. https://doi.org/10.1086/651229

34. Herrera F, Manchester SR, Koll R, Jaramillo CA Fruits of *Oreomunnea* (Juglandaceae) in the early Miocene of Panama Paleobotany and biogeography: A Festschrift for Alan Graham in his 80th year: Monographs in systematic botany from the Missouri Botanical Garden. 2014; 128:124–133.

35. Jud NA, Nelson CW, Herrera F. Fruits and wood of *Parinari* from the early Miocene of Panama and the fossil record of Chrysobalanaceae. American Journal of Botany. 2016; 103(2):277–289. https://doi.org/10.3732/ajb.1500425

36. Nelson CW, Jud NA Biogeographic implications of *Mammea paramericana* sp. nov. from the lower Miocene of Panama and the evolution of Calophyllaceae International Journal of Plant Sciences. 2017; 178(3):241–257. https://doi.org/10.1086/689618

37. Jud NA, Nelson CW. A liana from the lower Miocene of Panama and the fossil record of Connaraceae. American Journal of Botany. 2017; 104(5):685–693. https://doi.org/10.3732/ajb.1700080

38. Herrera F, Carvalho MR, Jaramillo CA, Manchester SR. 19-Million-Year-Old Spondioid Fruits from Panama Reveal a Dynamic Dispersal History for Anacardiaceae. International Journal of Plant Sciences. 2019; 180(6):479–492. https://doi.org/10.1086/703551

39. Berry EW. Contributions to the geology and paleontology of the Canal Zone, Panama and geologically related areas in Central America and the West Indies United States National Museum Bulletin. 1918; 103:15–44.

40. Rodríguez-Reyes O, Falcon-Lang HJ, Gasson P, Collinson ME, Jaramillo CA. Fossil woods (Malvaceae) from the lower Miocene (early to mid-Burdigalian) part of the Cucaracha Formation of Panama (Central America) and their biogeographic implications. Review of Palaeobotany and Palynology 2014; 209:11–34. https://doi.org/10.1016/j.revpalbo.2014.05.006

41. Rodríguez-Reyes O, Gasson P, Falcon-Lang HJ, Collinson ME. Fossil legume woods of the Prioria-clade (subfamily Detarioideae) from the lower Miocene (early to mid-Burdigalian) part of the Cucaracha Formation of Panama (Central America) and their systematic and palaeoecological implications Review of Palaeobotany and Palynology. 2017; 246:44–61. https://doi.org/10.1016/j.revpalbo.2017.06.005

42. Rodríguez-Reyes O, Gasson P, Thornton C, Falcon-Lang HJ, Jud NA. *Panascleroticoxylon crystallosa* gen. et sp. nov.: a new Miocene malpighialean tree from Panama. IAWA Journal. 2017; 38(4):437–455. https://doi.org/10.1163/22941932-20170178

43. Jaramillo CA, Moreno E, Ramírez V, da Silva-Caminha SAF, de la Barrera A, de la Barrera A, et al. Palynological record of the last 20 million years in Panama. Paleobotany and biogeography: A festschrift for Alan Graham in his 80th year. 2014; 128:134–251.

44. Joy KW, Willis AJ, Lacey WS. A rapid cellulose peel technique in palaeobotany. Annals of Botany. 1956; 20(80):635–637.

45. Enquist BJ, Condit R, Peet RK, Schildhauer M, Thiers BM. Cyberinfrastructure for an integrated botanical information network to investigate the ecological impacts of global climate change on plant biodiversity. PeerJ Inc.; 2016. e2615v2. Available from: https://peerj.com/preprints/2615.

46. Maitner BS, Boyle B, Casler N, Condit R, Donoghue J, Durán SM, et al. The bien r package: A tool to access the Botanical Information and Ecology Network (BIEN) database. Methods in Ecology and Evolution. 2018; 9(2):373–379. https://doi.org/10.1111/2041-210X.12861

47. Graham A. Studies in neotropical paleobotany. II. The Miocene communities of Veracruz, Mexico. Annals of the Missouri Botanical Garden. 1976; 63(4):787–842. https://doi.org/10.2307/2395250

48. Graham A. Tropical American Tertiary floras and paleoenvironments: Mexico, Costa Rica, and Panama. American Journal of Botany. 1987; 74(10):1519–1531. https://doi.org/10.1002/j.1537-2197.1987.tb12143.x

49. Graham A. Studies in Neotropical Paleobotany. X. The Pliocene Communities of Panama–Composition, Numerical Representations, and Paleocommunity Paleoenvironmental Reconstructions. Annals of the Missouri Botanical Garden. 1991; 78(2):465–475. https://doi.org/10.2307/2399574

50. Chavez RP, Rzedowski J. Estudio palinológico de las floras fósiles del Mioceno inferior y principios del Mioceno medio de la región de Pichucalco, Chiapas, México. Acta Botánica Mexicana. 1993; 24:1–96.

51. Hendy AJW. Spatial and stratigraphic variation of marine paleoenvironments in the middle-upper Miocene Gatun Formation, Isthmus of Panama. PALAIOS. 2013; 28(4):210–227. https://doi.org/10.2110/palo.2012.p12-024r

52. Graham A. Studies in Neotropical Paleobotany. IV. The Eocene communities of Panama. Annals of the Missouri Botanical Garden. 1985; 72(3):504–534. https://doi.org/10.2307/2399101

53. GBIF.org [11 January 2021] GBIF Occurrence Download https://doi.org/10.15468/dl.7yscgt

54. Gildenhuys E, Ellis AG, Carroll SP, Le Roux JJ. The ecology, biogeography, history and future of two globally important weeds: *Cardiospermum halicacabum* Linn. and *C. grandiflorum* NeoBiota. 2013; 19:45–65. https://doi.org/10.3897/neobiota.19.5279

55. Gildenhuys E, Ellis AG, Carroll SP, Le Roux JJ. Combining natal range distributions and phylogeny to resolve biogeographic uncertainties in balloon vines (*Cardiospermum*, Sapindaceae). Diversity and Distributions. 2015; 21(2):163–174. https://doi.org/10.1111/ddi.12261

56. Wheeler EA, Baas P, Gasson PE, et al. IAWA list of microscopic features for hardwood identification. IAWA Journal. 1989.

57. Carlquist S. Wood anatomy of Nepenthaceae. Bulletin of the Torrey Botanical Club. 1981; 108 (3):324–330. https://doi.org/10.2307/2484711

58. Carlquist S. Observations on Functional Wood Histology of Vines and Lianas. Aliso: A Journal of Systematic and Evolutionary Botany. 1985; 11(2):139–157. https://doi.org/10.5642/aliso.19851102.03

59. Carlquist S. Anatomy of vine and liana stems. In: The Biology of Vines. Cambridge University Press; 1991. p. 53–72.

60. Scrucca L, Fop M, Murphy TB, Raftery AE. mclust 5: clustering, classification and density estimation using Gaussian finite mixture models. The R journal. 2016; 8(1):289. https://doi.org/10.32614/RJ-2016-021

61. R Core Team. R: A Language and Environment for Statistical Computing. Vienna, Austria; 2020. Available from: https://www.R-project.org/.

62. Schneider CA, Rasband WS, Eliceiri KW. NIH Image to ImageJ: 25 years of image analysis. Nature methods. 2012; 9(7):671–675. https://doi.org/10.1038/nmeth.2089

63. Ellis B, Daly DC, Hickey LJ, Mitchell JD, Johnson KR, Wilf P, et al. Manual of Leaf Architecture. First edition ed. Ithaca, NY: Cornell University Press; 2009.

64. Chery JG, da Cunha Neto IL, Pace MR, Acevedo-Rodríguez P, Specht CD, Rothfels CJ. Wood anatomy of the neotropical liana lineage *Paullinia* L. (Sapindaceae). IAWA Journal. 2020; 41(3):278–300. https://doi.org/10.1163/22941932-bja10027

65. Patel RN. A comparison of the anatomy of the secondary xylem in roots and stems. Holzforschung-International Journal of the Biology, Chemistry, Physics and Technology of Wood. 1965; 19(3):72–79.

66. Bastos CL. Decifrando raízes e caules de lianas de Sapindaceae: diversidade macroscópica e conexões vasculares [PhD Thesis]. Universidade de São Paulo; 2015.

67. Ronquist F, Teslenko M, Van Der Mark P, Ayres DL, Darling A, Höhna S, et al. MrBayes 3.2: efficient Bayesian phylogenetic inference and model choice across a large model space. Systematic biology. 2012; 61(3)539–542. https://doi.org/10.1093/sysbio/sys029 PMID: 22357727

68. Letunic I, Bork P. Interactive Tree Of Life (iTOL) v4: recent updates and new developments. Nucleic Acids Research. 2019; 47(W1):W256–W259. https://doi.org/10.1093/nar/gkz239

69. Bouckaert R, Heled J, Kühnert D, Vaughan T, Wu C-H, Xie D, et al. BEAST 2: a software platform for Bayesian evolutionary analysis. PLoS Comput Biol. 2014; 10(4):e1003537. https://doi.org/10.1371/journal.pcbi.1003537 PMID: 24722319

70. Höhna S, Landis MJ, Heath TA, Boussau B, Lartillot N, Moore BR, et al. RevBayes: Bayesian phylogenetic inference using graphical models and an interactive model-specification language. Systematic biology. 2016; 65(4):726–736. https://doi.org/10.1093/sysbio/syw021 PMID: 27235697

71. Ewers FW, Fisher JB, Chiu S-T. A survey of vessel dimensions in stems of tropical lianas and other growth forms. Oecologia 1990; 84(4):544–552. https://doi.org/10.1007/BF00328172

72. Ewers FW, Carlton MR, Fisher JB, Kolb KJ, Tyree MT. Vessel diameters in roots versus stems of tropical lianas and other growth forms. IAWA Journal. 1997; 18(3):261–279. https://doi.org/10.1163/22941932-90001490

73. Rosell JA, Olson ME. Do lianas really have wide vessels? Vessel diameter–stem length scaling in non-self-supporting plants. Perspectives in Plant Ecology, Evolution and Systematics. 2014; 16(6):288–295. https://doi.org/10.1016/j.ppees.2014.08.001

74. MacGinitie HD. An early middle Eocene flora from the Yellowstone-Absaroka volcanic province, northwestern Wind River Basin, Wyoming. First edition ed. Berkeley: University of California Press; 1974.

75. Allen SE. The uppermost Lower Eocene Blue Rim flora from the Bridger Formation of Southwestern Wyoming: Floristic Composition, Paleoclimate, and Paleoecology [PhD Thesis]. University of Florida. Gainesville, FL; 2017.

76. Lesquereux L. Contributions to the flora of the Western Territories III. The Cretaceous and Tertiary floras. Rept US Geol Surv Terr. 1883; 8:1–283.

77. MacGinitie HD. Fossil plants of the Florissant beds, Colorado. vol. 599 of Carnegie Institution of Washington Publication. Carnegie Institution of Washington; 1953.

78. Becker HF. Oligocene plants from the upper Ruby River Basin, southwestern Montana. vol. 82. Geological Society of America; 1961.

79. Lielke K, Manchester SR, Meyer H, and others. Reconstructing the environment of the northern Rocky Mountains during the Eocene/Oligocene transition: constraints from the palaeobotany and geology of south-western Montana, USA. Acta palaeobotanica. 2012; 52(2):317–358.

80. Knowlton FH. Revision of the flora of the Green River formation, with descriptions of new species; 1923. 131–F. Available from: http://pubs.er.usgs.gov/publication/pp131F.

81. MacGinitie HD. The Eocene Green River flora of northwestern Colorado and northeastern Utah. University of California Press; 1969.

82. Brown RW. Additions to the flora of the Green River Formation. US Geological Survey Professional Paper. 1929;154:279–292.

83. Brown RW. The recognizable species of the Green River flora. United States. Government Printing Office. 1934.

84. Johnson KR, Plumb C. Common plant fossils from the Green River Formation at Douglas Pass, Colorado, and Bonanza, Utah. In: The Green River Piceance Creek and eastern Unita Basin: Grand Junction Colorado. Grand Junction Geological Society. 1995; p. 121–130.

85. Wolfe JA, Wehr W. Middle Eocene dicotyledonous plants from Republic, northeastern Washington. US Geological Survey Bulletin 1597. 1987;p. 1–25.

86. McClain AM, Manchester SR. *Dipteronia* (Sapindaceae) from the Tertiary of North America and implications for the phytogeographic history of the Aceroideae. American Journal of Botany. 2001; 88 (7):1316–1325. https://doi.org/10.2307/3558343

87. Fittipaldi FC, Simões MG, Giulietti AM, Pirani JR. Fossil plants from the Itaquaquecetuba Formation (Cenozoic of the São Paulo Basin) and their possible paleoclimatic significance. Boletim IG-USP Publicação Especial. 1989; 7:183–203.

88. Dos Santos D, Garcia M, Saad A, Bistrichi C. Itaquaquecetuba Formation Palynostratigraphy, São Paulo Basin, Brazil. Revista Brasileira de Paleontologia. 2010; 13:205–220.

89. Duarte L, Pimentel de Rezende-Martins A. Contribuíção ao conhecimento da flora Cenozóica do Brasil: Jazigo Vargem Grande do Sul, SP: série taubaté. I. Anais da Academia Brasileira de Ciências. 1983; 55(1):109–121.

90. Dos Santos MA, Bernardes-de Oliveira MEC. Taxonomia da tafoflora Neogena da Formação Rio Claro, Jaguariuna, Estado de Sao Paulo, Brasil. Revista Brasileira de Paleontolgia. 2013; 16(3):465–486. https://doi.org/10.4072/rbp.2013.3.07

91. Edwards WN, Wonnacott F. Sapindaceae. In: Fossilum Catalogus 2, Plantae. pars. 14. W. Junk; 1928 p. 1–84.

92. Cunha Neto IL, Martins FM, Somner GV, Tamaio N. Secretory structures in stems of five lianas of Paullinieae (Sapindaceae): morphology and histochemistry Flora. 2017; 235:29–40

93. Pellissari LCO, Barros CF, Medeiros H, Tamaio N. Cambial patterns of *Paullinia* (Sapindaceae) in southwestern Amazonia, Brazil. Flora. 2018; 246-247:71–82. https://doi.org/10.1016/j.flora.2018.07.002

94. Metcalfe CR, Chalk L. Anatomy of the Dicotyledons, Vols. 1 & 2. Clarendon Press; 1950.

95. Manchester SR. Update on the megafossil flora of Florissant, Colorado. Denver Museum of Nature and Science. 2001; 4:137–161.

96. Jaramillo C, Rueda MJ. A Morphological Electronic Database of Cretaceous-Tertiary and Extant Pollen and Spores from Northern South America, v. 2020. Smithsonian Tropical Research Institute. http://biogeodb.stri.si.edu/jaramillosdb/web/morphological/ (accessed 18 Jan 2021)

97. Ferrucci MS, Anzotegui LM. El polen de Paullinieae (Sapindaceae). Bonplandia. 1993;p. 211–243.

98. Van der Ham R, Tomlik A. *Serjania* pollen and the origin of the tribe Paullinieae (Sapindaceae). Review of Palaeobotany and Palynology. 1994; 83(1-3):43–53. https://doi.org/10.1016/0034-6667(94)90056-6

99. Montes C, Cardona A, McFadden R, Morón SE, Silva CA, Restrepo-Moreno S, et al. Evidence for middle Eocene and younger land emergence in central Panama: Implications for Isthmus closure. Geological Society of America Bulletin. 2012; 124(5-6):780–799. https://doi.org/10.1130/B30528.1

100. Ramírez DA, Foster DA, Min K, Montes C, Cardona A, Sadove G. Exhumation of the Panama basement complex and basins: Implications for the closure of the Central American seaway. Geochemistry, Geophysics, Geosystems. 2016; 17(5):1758–1777. https://doi.org/10.1002/2016GC006289

101. Leopold EB, MacGinitie HD. Development and affinities of Tertiary floras in the Rocky Mountains. In: Floristics and Paleofloristics of Asia and Eastern North America. Elsevier Publishing Company; 1972. p. 147–200.

102. Gray J. Temperate pollen genera in the Eocene (Claiborne) flora, Alabama. Science. 1960; 132 (3430):808–810. https://doi.org/10.1126/science.132.3430.808

103. Muller J. Fossil pollen records of extant angiosperms. The Botanical Review. 1981; 47(1):1–142. https://doi.org/10.1007/BF02860537

104. Bellonzi TK, Dutra FV, de Souza CN, Rezende AA, Gasparino EC. Pollen types of Sapindaceae from Brazilian forest fragments: apertural variation. Acta Botanica Brasilica. 2020; 34(2):327–341. https://doi.org/10.1590/0102-33062020abb0022

105. Carlquist S. Comparative Wood Anatomy: Systematic, Ecological, and Evolutionary Aspects of Dicotyledon Wood. Springer Science & Business Media; 2001.

106. Gentry AH. Distribution and evolution of climbing plants. In: The Biology of Vines. Cambridge University Press; 1991. p. 3–49.

107. Schnitzer SA, Bongers F. The ecology of lianas and their role in forests. Trends in Ecology & Evolution. 2002; 17(5):223–230. https://doi.org/10.1016/S0169-5347(02)02491-6

108. Schnitzer SA. A mechanistic explanation for global patterns of liana abundance and distribution. The American Naturalist. 2005; 166(2):262–276. https://doi.org/10.1086/431250

109. Van Der Heijden GMF, Phillips OL. Environmental effects on Neotropical liana species richness. Journal of Biogeography. 2009; 36(8):1561–1572. https://doi.org/10.1111/j.1365-2699.2009.02099.x
